# m^6^A RNA modification: a central epitranscriptomic switch orchestrating oncogenic signaling and glycolytic reprogramming in cancer

**DOI:** 10.3389/fcell.2026.1864321

**Published:** 2026-07-03

**Authors:** Yonggang Guo, Ruilong Kou, Zhizhong Huang, Zhihua Chen, Heng Yun

**Affiliations:** 1 Pingdingshan University, Pingdingshan, China; 2 Department of Gastrointestinal Surgery, Affiliated Hospital of Guilin Medical University, Guilin, China; 3 School of Pharmacy, Guizhou University of Traditional Chinese Medicine, Guiyang, China; 4 Department of Gastrointestinal Surgery II, The First Affiliated Hospital of Fujian Medical University, Fuzhou, China; 5 Department of General Surgery, The Third Affiliated Hospital of Gansu University of Traditional Chinese Medicine, Baiyin, China

**Keywords:** epitranscriptomics, glycolysis, M6A, signaling pathway, tumor

## Abstract

N6-methyladenosine (m6A) is the most widespread, abundant, and conserved post-transcriptional modification in eukaryotic RNA, and it participates in the regulation of various biological processes, especially playing a crucial role in tumorigenesis and progression. During tumor progression, abnormal expression of m6A regulatory proteins often leads to dysregulation of m6A modification levels, thereby affecting tumor pathophysiology. Recent studies have shown that in various tumor types, m6A modifications on target mRNAs and non-coding RNA transcripts can regulate the activity of various oncogenic signaling pathways; moreover, m6A modifications can also regulate the tumor glycolysis process through multiple molecular mechanisms, thereby affecting the proliferation, invasion, and metastasis of tumor cells and other biological behaviors. Most existing reviews focus only on the unidirectional regulatory relationships among m6A modification, oncogenic signaling, and glycolysis, while overlooking the crosstalk among the three. To address this gap, this review systematically summarizes the regulatory effects of m6A modifications on key glycolytic enzymes and various cancer signaling pathways, examines in depth the molecular mechanisms by which the three cooperatively participate in tumorigenesis and progression, comprehensively dissects the bidirectional crosstalk among the three core functional modules within this network, and further proposes a self-stabilizing “m6A–signaling–glycolysis closed-loop regulatory network,” and provides future research directions for this field. It offers theoretical references for related basic research and clinical diagnosis and treatment.

## 1 Introduction

Since Desrosiers et al. first confirmed the existence of N6-methyladenosine (m6A) in mRNA in the 1970s (Desrosiers et al., 1974), this modification has emerged as a central focus in the field of post-transcriptional RNA regulation. The m6A modification affects almost the entire process of RNA metabolism, and plays an important regulatory role in key biological processes such as alternative splicing of pre-mRNA, miRNA processing, mRNA stability, and mRNA degradation (Kretschmer et al., 2018). A large number of studies have confirmed that abnormal elevation of m6A modification levels and dysregulation of related regulatory proteins are associated with various human diseases and are particularly closely related to cancer development and progression (Chen et al., 2018; Chang et al., 2020). The m6A modification affects the malignant progression of tumors by influencing tumor proliferation, glycolysis, and cell apoptosis; at the same time, it also participates in regulating the tumor immune environment, T cell homeostasis, the development of the immune system, and the induction of immune responses (Huang et al., 2020). More importantly, m6A modification can serve as a key molecular link connecting three major biological processes—epigenetic regulation, cellular signal transduction, and energy metabolism. It can both target upstream oncogenic signaling pathways to alter the biological state of tumor cells and indirectly regulate glycolytic metabolism and remodel the tumor microenvironment, running through the entire process of tumorigenesis and progression.

**FIGURE 1:**
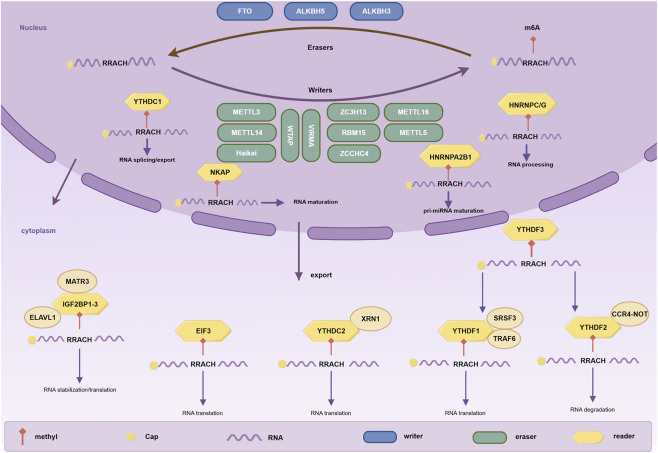
Components of m6A.

m6A RNA methylation is a dynamic process controlled by m6A writers, erasers, and readers, and it is reversible (Figure 1). The key enzymes mainly include m6A methyltransferases (writers), m6A demethylases (erasers), and m6A RNA-binding proteins (readers). m6A methyltransferases, also known as “writers,” are responsible for installation and include methyltransferase-like 3 (METTL3), methyltransferase-like 14 (METTL14), and Wilms’ tumor 1-associated protein (WTAP), among others. m6A demethylases (the “erasers”) promote the conversion of m6A to N6-hydroxymethyladenosine and N6-formyladenosine, ultimately hydrolyzing into adenosine, with the main members including fat mass and obesity-associated protein (FTO) and AlkB homolog 5 (ALKBH5) (Shi H. et al., 2019). “Readers” mainly exert their effects by regulating the rate of RNA degradation to enhance RNA translation efficiency or affect RNA stability (Qiu et al., 2023), including the YTH domain-containing family proteins, comprising YTH N6-methyladenosine RNA-binding proteins (YTHDF1/2/3) and YTH domain-containing proteins (YTHDC1/2), and insulin-like growth factor 2 mRNA-binding proteins 1/2/3 (IGF2BP1/2/3), etc.

## 2 m6A regulators

### 2.1 Writers

METTL3 was first identified as an m6A methyltransferase in the 1990s, and it is widely involved in various physiological processes such as embryonic development, cell reprogramming, and T-cell homeostasis (Li H.-B. et al., 2017). Recent studies have shown that abnormal expression of METTL3 is also considered an important factor contributing to cancer, promoting the development of colorectal cancer (Chen H. et al., 2021) and pancreatic cancer (Zhang et al., 2019); its abnormal expression has likewise been confirmed in human papillomavirus-positive head and neck squamous cell carcinoma (Yu et al., 2022). In gastric cancer it promotes tumor angiogenesis and liver metastasis (Wang et al., 2020c), and in pancreatic cancer, METTL3-mediated m6A modification of Yes-associated protein 1 (YAP1) promotes liver metastasis (Ni et al., 2021). Methyltransferase-like 5 (METTL5) is another important member of the conserved METTL family, with a function independent of the m6A methyltransferase complex (MTC) and a potential role in translation regulation (van Tran et al., 2019). It synergizes with its cofactor TRMT112 to drive pancreatic cancer progression (Lin C. et al., 2023) and accelerates the growth of breast cancer cells (Rong et al., 2020); its high expression is closely linked to the histological grade, stage, and overall survival of hepatocellular carcinoma (HCC) (Peng H. et al., 2022), and its abnormal expression is associated with the development of lung adenocarcinoma (LUAD) (Sun et al., 2020a). METTL14 is another key enzyme of the methyltransferase complex, interacting with METTL3 to form a stable heterodimer and thereby widely participating in the regulation of tumor proliferation, invasion, migration, metastasis, and drug resistance. It promotes leukemogenesis by regulating its mRNA targets such as MYB and MYC (Weng et al., 2018) and weakens the gemcitabine resistance of pancreatic cancer cells upon knockout (Zhang C. et al., 2021), while its abnormal expression is associated with the occurrence and poor prognosis of breast cancer (Gong et al., 2020); by contrast, it suppresses the migration, invasion, and metastasis of colorectal cancer cells *in vitro* (Chen X. et al., 2020), correlates negatively with the stage of renal cell carcinoma (Wang Q. et al., 2019), and promotes the tumorigenicity and chemotherapy resistance of osteosarcoma (Li et al., 2022b). Methyltransferase-like 16 (METTL16) is an m6A methyltransferase that functions independently of the MTC; it is widely distributed in the nucleus and cytoplasm and can recognize and bind to various RNA sites, providing a functional basis for its participation in tumorigenesis and progression (Aoyama et al., 2020; Ruszkowska, 2021). It drives the development of acute myeloid leukemia (AML) and the self-renewal of AML stem cells (Han et al., 2023), promotes breast cancer progression (Ye et al., 2023a), and is associated with HCC progression and poorer survival (Dai et al., 2022), whereas in ovarian cancer it instead inhibits the proliferation, migration, and invasion of cancer cells (Li C. et al., 2023).

Hakai was initially identified as the E3 ubiquitin ligase of the E-cadherin complex, mediating the ubiquitination, endocytosis, and degradation of E-cadherin in lysosomes (Bonazzi et al., 2008); it also participates in regulating cell proliferation and promotes the expression of cancer-related genes, playing an important role in tumor development (Rodríguez-Rigueiro et al., 2011). RNA-binding motif protein 15 (RBM15) is an important regulator of RNA m6A modification and a regulatory subunit of the MTC, promoting carcinogenesis in laryngeal squamous cell carcinoma (Wang X. et al., 2021) and the progression of HCC (Cai et al., 2021) and colorectal cancer (Zhang et al., 2022d); its expression correlates positively with tumor-infiltrating immune cells in pancreatic cancer, where overexpression promotes cell proliferation (Zhao Z. et al., 2022). KIAA1429 (VIRMA) is the largest known component of the MTC and can affect the transcription and translation of RNAs related to cell growth and proliferation, ultimately leading to abnormal cell proliferation and carcinogenesis (Hong, 2017); it promotes the progression and metastasis of liver cancer (Lan et al., 2019), the development of gastric cancer (Yang D. et al., 2021) and LUAD (Zhao and Xie, 2021), the drug resistance of non-small cell lung cancer (NSCLC) (Tang et al., 2021), and the proliferation and metastasis of colorectal cancer cells (Li Y. et al., 2022). WTAP is one of the core complex components that catalyze m6A modification; it can stabilize METTL3 and METTL14 and is involved in alternative splicing and cell cycle regulation (Chen et al., 2019), reducing the apoptosis of bladder cancer cells (Wei W. et al., 2021), promoting the malignant phenotype of NSCLC (Weng et al., 2020) and the migration and invasion of glioblastoma (GBM) cells (Jin et al., 2012), and increasing the proliferation of AML cells (Bansal et al., 2014). The zinc finger CCCH domain protein 13 (ZC3H13), as an m6A methyltransferase, primarily tethers RBM15, WTAP, and VIRMA together to assemble the m6A methyltransferase complex (Wen et al., 2018); it contributes to the tumorigenesis and progression of breast cancer as a prognostic marker (Gong et al., 2020) and is associated with the proliferative and invasive capacities of colorectal cancer cells (Zhu et al., 2019). ZCCHC4 is a newly discovered m6A “writer” that is abnormally expressed in various tumors and plays an important regulatory role in tumor progression and treatment: its downregulation inhibits the proliferation of liver cancer cells (Ma et al., 2019), whereas its upregulation is associated with poor prognosis and chemotherapy resistance in liver cancer (Zhu H. et al., 2022); it is further linked to the progression of colon cancer (Chen et al., 2024a) and small cell lung cancer (Zhang Z. et al., 2021).

### 2.2 Erasers

FTO is the first m6A demethylase to be discovered, which can remove the m6A modification from mRNA *in vitro* and in cells, thereby regulating the stability, RNA processing, splicing, localization, translocation, surveillance, decay, and translation of downstream target mRNAs (Azzam et al., 2022). Numerous studies have demonstrated that in human cancer cells, FTO is located in the cytoplasm and promotes cancer progression. High expression of FTO increases the proportion of stem cell-like cells in HCC (Bian et al., 2021); abnormal expression of FTO can regulate the proliferation, apoptosis, and migration abilities of breast cancer cells (Xu et al., 2020); in addition, abnormal expression of FTO is associated with colon cancer metastasis (Ruan et al., 2021); FTO-mediated demethylation activates the MALAT1/miR-384/MAL2 cascade, promoting the development of bladder cancer (Tao et al., 2021). ALKBH5 is the second RNA demethylase discovered that uses m6A as the sole known substrate, and it is widely involved in various biological processes such as RNA metabolism, cell proliferation, apoptosis, development, stress response, and tumorigenesis and progression (Fang et al., 2025). Current studies have shown that ALKBH5 can maintain the tumorigenicity of GBM stem-like cells by maintaining the expression of forkhead box protein M1 (FOXM1) (Zhang et al., 2017); ALKBH5 is abnormally overexpressed in AML and is associated with poor prognosis (Wang et al., 2020a); it promotes the growth, invasion, and metastasis of HCC tumor cells (Chen Y. et al., 2020); ALKBH5 can inhibit the proliferation of bladder cancer cells (Yu et al., 2021a), and in breast cancer, ALKBH5 promotes the self-renewal of cancer stem cells by mediating the methylation of NANOG mRNA (Zhang et al., 2016).

### 2.3 Readers

#### 2.3.1 YTH family

m6A readers are involved in many RNA processes, including mRNA splicing, nuclear export, translation, and decay during post-transcriptional regulation. Based on their structural features, they can be broadly grouped into the YTH family, the IGF2BP family, and other RNA-binding proteins. The YTH N6-methyladenosine RNA-binding protein (YTHDF) family, consisting of YTHDF1, YTHDF2, and YTHDF3, represents a group of m6A readers that exhibit different functions: YTHDF1 enhances the translation of m6A-modified mRNA (Yang C. et al., 2020), YTHDF2 accelerates mRNA turnover and degradation, and YTHDF3 has dual regulatory functions, simultaneously promoting mRNA translation and degradation (Zaccara and Jaffrey, 2020). YTHDF1 is one of the main m6A readers; it interacts with initiation factors to promote translation in the cytoplasm and is involved in regulating gene expression related to cancer, cell proliferation, migration and invasion, inflammation, immunity, and autophagy (Chen et al., 2022a). YTHDF1 is closely related to shorter progression-free survival in NSCLC patients (Wen C. et al., 2024); in breast cancer, its high expression promotes the metastasis, invasion, and angiogenesis of tumor cells (Chen et al., 2022b); it may also be related to immune evasion and drug resistance, as knocking down YTHDF1 significantly increases the sensitivity of colorectal cancer cells to fluorouracil and oxaliplatin (Nishizawa et al., 2018), and its high expression enhances the drug resistance of ovarian cancer cells (Hao et al., 2021). YTHDF2 is the first discovered m6A binding protein, mainly distributed in the cytoplasm, with the core function of promoting mRNA degradation and reducing the stability of target transcripts (Jiang X. et al., 2021). It plays a carcinogenic role in most cancer types: YTHDF2 is highly expressed in prostate cancer and negatively correlated with miR-493-3p (Li et al., 2018), promotes the stem cell phenotype and metastasis of HCC (Zhang et al., 2020), accelerates the progression of gastric cancer (Yang H. et al., 2022), and promotes metastasis through epithelial-mesenchymal transition (EMT) in bladder cancer (Zhang et al., 2023b), whereas it inhibits the progression of breast cancer by targeting EGFR (Zhong et al., 2019). YTHDF3 is an important m6A reader, mainly located in the cytoplasm, that can interact with YTHDF1 to enhance mRNA translation and with YTHDF2 to affect mRNA degradation and stability (Li A. et al., 2017). It promotes lymph node metastasis in cervical cancer (Zhong et al., 2024), the proliferation and migration of ocular melanoma (Xu Y. et al., 2022), metastasis in HCC (Wang et al., 2020b) and brain metastasis in breast cancer (Chang et al., 2020), and the tumorigenesis and progression of colorectal cancer (Ni et al., 2019). YTH domain-containing protein 1 (YTHDC1) is mainly located in the nucleus and regulates mRNA splicing, nuclear export, and stability (Shima et al., 2017); it mediates immune escape in NSCLC (Liu et al., 2021e) and is crucial for maintaining the stability of AML target mRNAs and cell survival (Cheng et al., 2021). YTH domain-containing protein 2 (YTHDC2) regulates mRNA translation and stability by recognizing m6A modifications (He et al., 2020); its expression is closely related to the tumorigenesis and progression of various tumors, increasing the risk of colon cancer metastasis (Tanabe et al., 2016), correlating with the prognosis of nasopharyngeal carcinoma (He et al., 2020), acting as an oncogene in pancreatic cancer (Fanale et al., 2014), and showing downregulation in lung cancer (Sun et al., 2020b). In addition, NKAP is an m6A reader that binds pre-mRNA and spliced mRNA by recruiting, activating, or stabilizing RNA processing factors (Burgute et al., 2014).

#### 2.3.2 IGF2BP family

The insulin-like growth factor 2 mRNA-binding protein (IGF2BP) family is another important family of m6A readers, consisting of IGF2BP1, IGF2BP2, and IGF2BP3, which regulate gene expression by recruiting mRNA stabilizers (Huang et al., 2018). The IGF2BP family plays a crucial role in tumorigenesis and progression, cancer progression, tumor metastasis, and invasion, and is a potential target for cancer treatment (Li Y. et al., 2019). IGF2BPs are highly expressed in renal cell carcinoma tissues (Ying et al., 2021); IGF2BP1 mediates the proliferation and metastasis of endometrial cancer cells (Xue et al., 2021) and activates SRF-dependent transcription to support tumor cell growth in ovarian, liver, and lung cancers (Müller et al., 2019); IGF2BP2 is abnormally highly expressed in liver cancer and promotes its proliferation (Pu et al., 2020), accelerates the progression of glioblastoma (GBM) by stabilizing CASC9 (Liu et al., 2021a), and is closely related to the tumorigenesis and progression of pancreatic cancer (Hu X. et al., 2020) and AML (Weng et al., 2022).

#### 2.3.3 Others (HNRNP and eIF3)

Heterogeneous nuclear ribonucleoproteins (HNRNPs) are another type of important m6A-related RNA-binding proteins, including HNRNPC, HNRNPG, and HNRNPA2B1, whose core function is to regulate the alternative splicing or processing of target transcripts (Alarcón et al., 2015). HNRNPC is mainly distributed in the nucleus and affects transcript abundance and alternative splicing by binding to m6A-modified mRNA (Lian et al., 2023); it has been identified as an oncogene that promotes malignant progression in esophageal cancer (Zhou Y. et al., 2023), gastric cancer (Huang et al., 2016), breast cancer (Wu et al., 2018), oral squamous cell carcinoma (OSCC) (Zhu W. et al., 2022), and papillary renal cell carcinoma (Wang et al., 2023f). HNRNPA2B1 is involved in various biological processes such as RNA transcription, stability, splicing, and translation (Liu and Shi, 2021); it increases the viability of pancreatic ductal adenocarcinoma cells (Barceló et al., 2014), promotes the development of colorectal cancer (Liu et al., 2022c), and enhances the malignant potential of ovarian cancer (Yang Y. et al., 2020), whereas in HCC it instead inhibits tumor progression (Wang et al., 2018). Among the eukaryotic translation initiation factors (eIFs), the eIF3 complex consisting of 13 subunits from eIF3a to eIF3m is the largest and most complex one (Browning et al., 2001); as an m6A reader, it regulates the translation efficiency of target mRNAs and participates in tumorigenesis (Shah et al., 2017). Abnormal expression of the eIF3 complex is closely related to the malignant transformation of various tumors, and overexpression of its subunits eIF3a, eIF3b, eIF3c, eIF3i, eIF3h, and eIF3m can promote malignant transformation (Yuan et al., 2014; Hershey, 2015); eIF3a is involved in the progression of cervical cancer (Dellas et al., 1998), esophageal cancer (Chen and Burger, 1999), lung cancer (Pincheira et al., 2001), ovarian cancer (Zhang et al., 2015), and pancreatic cancer (Wang et al., 2016). The regulatory effects and underlying mechanisms of m6A regulators in various tumors are summarized in Table 1.

**TABLE 1:** Regulation and mechanisms of m6A in tumors.

m6A	Tumor	Mechanism	References
METTL3	Colon cancer	Inhibits GLUT and glycolysis	Chen G. et al. (2021)
​	Pancreatic cancer	Regulates and stabilizes miR-25-3p expression	Zhang et al. (2019)
​	HNSCC	Negatively correlated with tumor immune cell infiltration	Yu et al. (2022)
​	Gastric cancer	Regulates HDGF mRNA stability and promotes tumor angiogenesis	Wang et al. (2020c)
​	LUAD	Increases m6A modification and expression of YAP1 mRNA	Ni et al. (2021)
METTL5	Pancreatic cancer	Cooperates with its cofactor TRMT112	Lin C. et al. (2023)
​	HCC	Associated with histological grade, tumor stage, and overall survival	Peng H. et al. (2022)
​	Breast cancer	Promotes translation of p70-S6K	Rong et al. (2020)
​	LUAD	Associated with lung cancer prognosis	Sun et al. (2020a)
METTL14	AML	Regulates expression of MYB and MYC	Weng et al. (2018)
​	Pancreatic cancer	Regulates gemcitabine resistance	Zhang C. et al. (2021)
​	Breast cancer	Strongly positively correlated with the Wnt signaling pathway antagonist APC	Gong et al. (2020)
​	CRC	Regulates SOX4 mRNA expression	Chen X. et al. (2020)
​	Renal cell carcinoma	Regulates PTEN mRNA expression	Wang Q. et al. (2019)
​	Osteosarcoma	Induces MN1 expression	Li H.-B. et al. (2022)
METTL16	AML	Regulates expression of BCAT1-2/BCAA	Han et al. (2023)
​	Ovarian cancer	Promotes MALAT1 degradation and upregulates β-catenin	Li C. et al. (2023)
​	Breast cancer	Enhances GPX4 expression	Ye et al. (2023a)
​	HCC	Reduces stability of RAB11B-AS1 transcripts	Dai et al. (2022)
RBM15	Laryngeal squamous cell carcinoma	Enhances TMBIM6 stability	Wang X. et al. (2021)
​	HCC	Regulates m6A modification of YES1 mRNA	Cai et al. (2021)
​	CRC	Regulates MyD88 expression	Zhang et al. (2022d)
​	Pancreatic cancer	Positively correlated with immune-infiltrating cells	Zhao Z. et al. (2022)
KIAA1429	HCC	Induces m6A methylation of GATA3 pre-mRNA	Lan et al. (2019)
​	Gastric cancer	Regulates glycolytic enzymes	Yang D. et al. (2021)
​	LUAD	Regulates MUC3A expression	Zhao and Xie (2021)
​	CRC	Regulates HK2 mRNA stability and glycolysis	Li Y. et al. (2022)
WTAP	Bladder cancer	Regulates expression of TNFα-induced protein 3 (TNFAIP3)	Wei W. et al. (2021)
​	NSCLC	miR-433-3p regulates WTAP	Weng et al. (2020)
​	GBM	Enhances EGFR phosphorylation	Jin et al. (2012)
​	AML	Increases proliferative capacity of AML cells	Bansal et al. (2014)
ZC3H13	Breast cancer	Positively correlated with the Wnt signaling pathway antagonist APC	Gong et al. (2020)
​	CRC	Inactivates the Ras-ERK signaling pathway	Zhu et al. (2019)
ZCCHC4	HCC	Impairs apoptosis by interacting with the novel lncRNA AL133467.2	Zhu H. et al. (2022)
​	Colon cancer	Downregulates GHRLOS to promote CRC	Chen et al. (2024a)
​	SCLC	Involved in tumor progression and drug resistance	Zhang Z. et al. (2021)
FTO	HCC	Enhances expression of SOX2, KLF4, and NANOG	Bian et al. (2021)
​	Breast cancer	Inhibits miR-181b-3p and upregulates ARL5B	Xu et al. (2020)
​	Colon cancer	Inhibits expression of metastasis-associated protein 1 (MTA1)	Ruan et al. (2021)
​	GBM	Regulates self-renewal of glioblastoma stem cells (GSCs)	Cui et al. (2017)
​	Bladder cancer	Activates MALAT1/miR-384/MAL2	Tao et al. (2021)
ALKBH5	GBM	Promotes FOXM1 expression	Zhang et al. (2017)
​	AML	Regulates post-transcriptional regulation of TACC3 and AXL	Wang et al. (2020a)
​	HCC	Causes post-transcriptional inhibition of LY6/PLAUR domain containing 1 (LYPD1)	Chen Y. et al. (2020)
​	Bladder cancer	Regulates m6A-CK2-mediated glycolysis and cisplatin sensitivity	Yu et al. (2021a)
​	Breast cancer	Regulates NANOG mRNA methylation	Zhang et al. (2016)
YTHDF1	NSCLC	Increases lysosomal cathepsins and triggers STING degradation	Wen C. et al. (2024)
​	Breast cancer	Regulates FOXM1 translation	Chen et al. (2022b)
​	CRC	Associated with sensitivity to fluorouracil and oxaliplatin	Nishizawa et al. (2018)
​	Ovarian cancer	Associated with cisplatin resistance	Hao et al. (2021)
YTHDF2	Prostate cancer	Interacts with miR-493-3p to regulate m6A levels	Li et al. (2018)
​	HCC	Regulates OCT4 expression	Zhang et al. (2020)
​	Gastric cancer	Regulates CBS mRNA stability	Yang H. et al. (2022)
​	Breast cancer	Regulates EGFR	Zhong et al. (2019)
​	Bladder cancer	Regulates EMT and stem cells	Zhang et al. (2023b)
YTHDF3	Cervical cancer	Regulates LRP6 and fatty acid metabolism	Zhong et al. (2024)
​	Melanoma	Regulates CTNNB1 translation	Xu Y. et al. (2022)
​	HCC	Regulates Zeb1 mRNA stability	Wang et al. (2020b)
​	Breast cancer	Regulates translation of GJA1 and EGFR	Chang et al. (2020)
​	CRC	Regulates degradation of lncRNA GAS5	Ni et al. (2019)
YTHDC1	NSCLC	Regulates PD-L1 ubiquitination and proteasomal degradation	Liu et al. (2021e)
​	AML	Forms nuclear condensates (nYACs)	Cheng et al. (2021)
YTHDC2	Colon cancer	Regulates HIF-1α translation	Tanabe et al. (2016)
​	Nasopharyngeal carcinoma	Regulates translation of insulin-like growth factor 1 receptor (IGF1R) mRNA and AKT signaling	He et al. (2020)
​	Pancreatic cancer	Acts as an oncogene in pancreatic cancer	Fanale et al. (2014)
​	NSCLC	Regulates tumor cell proliferation and migration	Sun et al. (2020b)
IGF2BPS	Renal cancer	Regulates S1PR3 mRNA stability	Ying et al. (2021)
​	Endometrial cancer	Regulates SOX2 mRNA degradation	Xue et al. (2021)
​	HCC	Regulates FEN1 mRNA stability	Pu et al. (2020)
​	GBM	Regulates CASC9 stability	Liu et al. (2021a)
​	Pancreatic cancer	Regulates DANCR stability	Hu X. et al. (2020)
​	AML	Promotes self-renewal of leukemia stem cells	Weng et al. (2022)
HNRNPC	Esophageal cancer	Regulates GLI2 mRNA stability	Zhou Y. et al. (2023)
​	Gastric cancer	Associated with chemotherapy resistance	Huang et al. (2016)
​	Breast cancer	Regulates type I interferon response	Wu et al. (2018)
​	OSCC	Regulates ZEB1 mRNA stability	Zhu W. et al. (2022)
​	Papillary renal cell carcinoma	Regulates VEGFC secretion	Wang et al. (2023f)
HnRNPA2B1	PDAC	Interacts with KRAS	Barceló et al. (2014)
​	CRC	Regulates TCF7L2 mRNA stability	Liu et al. (2022c)
​	Ovarian cancer	Regulates Lin28B stability	Yang Y. et al. (2020)
​	HCC	Regulates p52 and p65 mRNA stability	Wang et al. (2018)
eIF3a	Cervical cancer	Serves as a potential tumor marker	Dellas et al. (1998)
​	Esophageal cancer	Serves as a potential tumor marker	Chen and Burger (1999)
​	Lung cancer	Serves as a potential tumor marker	Pincheira et al. (2001)
​	Ovarian cancer	Regulates xeroderma pigmentosum complementation group C (XPC) and p27	Zhang et al. (2015)
​	Pancreatic cancer	Significantly associated with tumor metastasis and TNM stage	Wang et al. (2016)

AML, acute myeloid leukemia; CRC, colorectal cancer; GBM, glioblastoma; HCC, hepatocellular carcinoma; HNSCC, head and neck squamous cell carcinoma; LUAD, lung adenocarcinoma; NSCLC, non-small cell lung cancer; OSCC, oral squamous cell carcinoma; PDAC, pancreatic ductal adenocarcinoma; SCLC, small cell lung cancer.

### 2.4 m6A and cancer clinical therapy

Given the key regulatory roles of m6A regulatory proteins in tumor progression, targeting these molecules has emerged as a highly promising direction for anti-tumor drug development (Zhang et al., 2025c). In hepatocellular carcinoma (Zeng et al., 2020) and bladder cancer (Han J. et al., 2019; Alasar et al., 2022), METTL3 regulates the expression of target transcripts in an m6A-dependent manner, thereby inducing malignant tumor phenotypes and cisplatin resistance, respectively; small-molecule inhibitors targeting FTO have shown marked anti-tumor activity in multiple cancer models (Huang et al., 2019), whereas ALKBH5 can modulate the cisplatin response of bladder cancer cells via the glycolytic pathway (Yu et al., 2021a); at the level of reader proteins, YTHDF1 can bind m6A-modified mRNAs of lysosomal proteases and thereby weaken the efficacy of anti-tumor immunotherapy (Han D. et al., 2019); several small-molecule inhibitors targeting YTHDF2 have also achieved considerable anti-tumor effects in preliminary studies (Zhang et al., 2025a).

### 2.5 m6A and immune regulation in cancer

A growing body of evidence highlights the indispensable role of m6A modification in regulating the homeostasis of tumor immune metabolism. Studies have shown that loss of METTL3 disrupts the homeostatic proliferation and differentiation of T cells (Li H.-B. et al., 2017); in addition, METTL3 maintains the suppressive function of Tregs in autoimmunity (Tong et al., 2018). FTO plays a crucial role in promoting melanoma tumorigenesis and resistance to anti-PD-1 therapy (Yang et al., 2019). IGF2BP1 participates in the glycolysis of tumor cells and plays an important role in remodeling the tumor microenvironment (TME) and driving immune escape (Zhang X.-L. et al., 2021). YTHDF2 exerts distinct functions in lymphoid and myeloid cells across different tumor contexts, it is the most abundant RNA-modifying protein in patient samples of B-cell malignancies and can regulate the oncogenic transformation and immune evasion of multiple B-cell cancers (Zhang et al., 2025a), and small molecules targeting YTHDF2 reduce ATP synthesis, representing a therapeutic candidate for B-cell malignancies (Chen et al., 2025).

## 3 m6A regulates glycolysis

**FIGURE 2:**
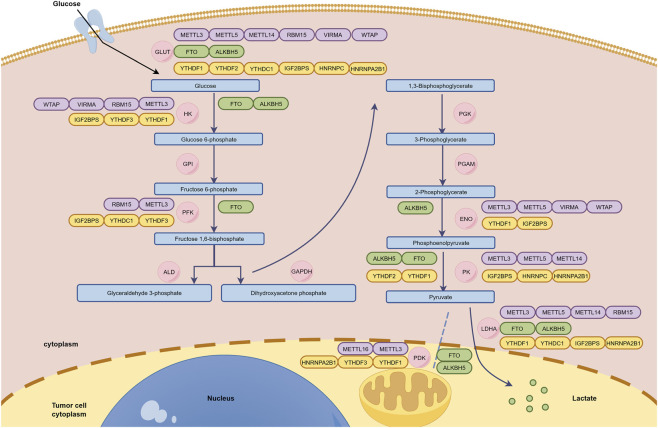
m6A regulates glycolysis.

Researchers have long recognized that altered metabolism is one of the hallmarks of cancer (Hanahan and Weinberg, 2011). Studies have confirmed that glycolysis can regulate the tumor microenvironment (TME) and the immune environment. Cancer cells can reduce the metabolic adaptation of tumor-infiltrating immune cells to inhibit the anti-tumor immune response (Guerra et al., 2020). Several enzymes drive glycolysis, including hexokinase (HK), phosphoglucose isomerase (GPI), phosphofructokinase (PFK), aldolase (ALDO), glyceraldehyde-3-phosphate dehydrogenase (GAPDH), phosphoglycerate kinase (PGK), phosphoglycerate mutase (PGM), enolase (ENO), pyruvate kinase (PK), pyruvate dehydrogenase (PDH), and lactate dehydrogenase (LDH). The pyruvate produced by glycolysis can directly enter the tricarboxylic acid cycle or be converted into lactate by LDH, and then lactate is transported out of the cell through monocarboxylate transporters. A large number of studies have shown that m6A methylation plays a key role in shaping hypoxic, low-glucose, and acidic TMEs (Figure 2), and can affect tumor proliferation, metastasis, and therapeutic response by regulating tumor glycolysis (Zhou et al., 2022).

### 3.1 m6A and HK2

HK plays a central role in glycolysis, catalyzing the conversion of glucose to glucose-6-phosphate in the first and rate-limiting irreversible reaction of glycolysis (Mathupala et al., 2006). There are five subtypes of HK in mammalian tissues (HK1, HK2, HK3, HK4, and HKDC1), with HK2 being the most active and abundant isoenzyme. Multiple m6A regulators converge on HK2, exerting opposing effects on glycolysis through m6A-dependent control of HK2 mRNA. On the one hand, several writers and readers stabilize or enhance HK2 expression to drive aerobic glycolysis and tumor progression. Among the writers, METTL3 stabilizes HK2 mRNA through the readers YTHDF1 and IGF2BP2, promoting aerobic glycolysis and cell growth in cervical and other cancers (Shen et al., 2020; Wang et al., 2020d); the circ-CTNNB1–RBM15 axis and VIRMA likewise enhance HK2 expression in an m6A-dependent manner, accelerating glycolysis in osteosarcoma and colorectal cancer (CRC) (Li Y. et al., 2022; Yang et al., 2023). WTAP is a particularly recurrent activator of HK2: it stabilizes HK2 mRNA or elevates its m6A level to accelerate the Warburg effect and tumor progression across gastric cancer, HCC, and diffuse large B-cell lymphoma (DLBCL) (Han et al., 2021a; Yu et al., 2021b; Niu et al., 2025). On the reader side, YTHDF1 increases the stability or translation of HK2 mRNA to promote the Warburg effect in esophageal and cervical cancers (Wang et al., 2020d; 2022), YTHDF3 promotes pancreatic cancer glycolysis and metastasis by reducing the stability of DICER1-AS1 (Hu Y. et al., 2022), and IGF2BP2/IGF2BP3 positively regulate HK2 mRNA stability to support glycolysis in OSCC and lung cancer (Shen et al., 2020; Xu K. et al., 2022; Li P. et al., 2024). On the other hand, the erasers FTO and ALKBH5 can act as tumor suppressors by downregulating HK2: FTO and ALKBH5 reduce HK2 expression through IGF2BP2 to inhibit colon cancer (Ye et al., 2023b), FTO overexpression lowers HK2 mRNA and protein levels to suppress cervical cancer (Liu et al., 2022a), and ALKBH5 represses HK2 via m6A-dependent regulation of UBR7 to suppress glycolysis in HCC (Zhao L. et al., 2022). Together, these findings establish HK2 as a common downstream node through which m6A writers, erasers, and readers bidirectionally regulate tumor glycolysis.

### 3.2 m6A and PKM2

As another key rate-limiting enzyme in glycolysis, PK catalyzes the conversion of phosphoenolpyruvate into pyruvate, the final rate-limiting step of glycolysis. Numerous studies have confirmed that the expression and function of PK, especially its isozyme pyruvate kinase M2 (PKM2), are also regulated by m6A methylation, thereby participating in the malignant progression of various tumors (Sun et al., 2011). m6A regulators modulate PKM2 through three broadly distinct routes: by altering its mRNA stability or translation, by controlling its protein degradation, and by shifting the PKM1/PKM2 splicing ratio. First, several writers and readers upregulate PKM2 to drive glycolysis. METTL3 enhances PKM2 expression in esophageal and lung cancers, either by reducing APC to elevate the β-catenin–c-Myc–PKM2 axis or by stabilizing SRPK1 in an IGF2BP2-dependent manner, and more broadly upregulates a panel of glycolytic genes (SLC2A1, PFKL, PGK1, ENO1, and PKM) to promote esophageal cancer progression (Wang W. et al., 2021; Wang et al., 2024a; Liu X.-S. et al., 2024); METTL5 likewise activates downstream glycolytic genes including PKM2 to promote HCC (Xia et al., 2023). On the reader side, YTHDF1 increases PKM2 abundance to promote glycolysis in breast cancer (Yao et al., 2022), and TNFAIP6 cooperates with HNRNPC to upregulate PKM2 in liver cancer (Duan et al., 2024). Second, a number of regulators suppress PKM2 by promoting its degradation or destabilizing its mRNA, often acting as tumor suppressors. METTL3 together with IGF2BP2 drives RNF183 transcription to enhance PKM2 protein degradation, and METTL14 promotes PKM2 ubiquitination via miR-29c-3p in triple-negative breast cancer (TNBC) (Wu H. et al., 2024). In other contexts, m6A regulators instead reshape PKM2 to favor malignancy: the METTL14/ALKBH5/IGF2BPs module stabilizes JMJD8 to enhance PKM2 enzymatic activity in CRC (Wu S. et al., 2023), and ALKBH5 knockdown upregulates circNRIP1 and thereby PKM2 in thyroid cancer (Ji et al., 2023). FTO shows context-dependent effects, promoting PKM2 translation through demethylation in HCC (Li J. et al., 2019) while, in liver cancer, reducing m6A on GLUT1 and PKM2 to attenuate YTHDF2-mediated mRNA decay (Wang et al., 2023b); conversely, YTHDF2 binding to MSS51 lowers the expression of LDHA, PFKP, and PKM (Jiao et al., 2024), and circFAM13B competitively binds IGF2BP1 to destabilize PKM2 mRNA and inhibit glycolysis in bladder cancer (Lv et al., 2023). The IGF2BP2–miR-34a-5p interplay further fine-tunes PKM2-and LDHA-dependent glycolysis (Hu C. et al., 2023). Third, the HNRNP family regulates PKM2 at the level of alternative splicing rather than abundance. hnRNPA1 and hnRNPA1B2 control PKM pre-mRNA splicing to lower the PKM1/PKM2 ratio, promoting the aerobic glycolysis required for tumor cell proliferation (David et al., 2010; Gao et al., 2021; Rihan and Sharma, 2024), while HNRNPC regulates PKM splicing through m6A modification to promote papillary thyroid carcinoma (Rong et al., 2024). Collectively, these studies position PKM2 as a central glycolytic target on which m6A regulators converge through stability, degradation, and splicing mechanisms to bidirectionally shape tumor metabolism.

### 3.3 m6A and PDK

As a key regulatory enzyme between glycolysis and mitochondrial respiration, pyruvate dehydrogenase kinase (PDK) is also involved in tumor metabolic reprogramming and malignant progression. The PDK family has four subtypes (PDK1–4). Among them, PDK1 is a key metabolic enzyme that can prevent the flow of pyruvate into mitochondrial respiration and is closely related to tumor proliferation and metastasis (Dupuy et al., 2015). m6A regulators control glycolysis through different members of the PDK family. PDK4 is a recurrent target of the writers and their readers: in cervical cancer, METTL3 installs 5′UTR m6A on PDK4 and promotes its recognition by IGF2BP3 and YTHDF1, enhancing PDK4 mRNA stability and translation, whereas METTL3 deletion reduces PDK4 expression and PDK4 overexpression weakens METTL3-driven glycolysis (Li Z. et al., 2020); and METTL16 upregulates PDK4 via IGF2BP1-mediated enhancement of SOGA1 to promote CRC (Wei W. et al., 2024). FTO acts mainly on PDK1: the JPX–FTO interaction enhances FTO-mediated demethylation of PDK1 mRNA to promote glycolysis in GBM (Li et al., 2021e), while in breast cancer FTO upregulates PDK1 and promotes PD-L1 expression to regulate immune escape (Wang et al., 2024f). In addition, PDK2 is regulated indirectly: hnRNPA2B1 stabilizes p53 pre-mRNA by binding its 3′UTR m6A sites (Liu et al., 2021b), and p53 in turn downregulates PDK2 to modulate tumor glycolysis (Lacroix et al., 2020).

### 3.4 m6A and PFK

6-Phosphofructo-1-kinase (PFK1) is the second rate-limiting enzyme in the glycolytic pathway and exists in three different subtypes: PFKM, PFKL, and PFKP. 6-Phosphofructo-2-kinase/fructose-2,6-bisphosphatase 3 (PFKFB3) is an allosteric activator of PFK1, which can significantly enhance the enzymatic activity of PFK1 (Saunier et al., 2016). Both PFK1 and PFKFB3 can serve as targets for regulating aerobic glycolysis and improving the chemotherapy efficacy of primary and metastatic tumors (Ni et al., 2024). m6A writers and erasers regulate glycolysis by acting on different PFK subtypes. METTL3 promotes m6A methylation of key glycolytic genes including PFKFB3, and acts through IGF2BP2 on HK2, PFK, and PKM; in gastric cancer it stabilizes PFKFB3 to reduce pyroptosis and promote glycolysis (Li Y. et al., 2023; Ouyang et al., 2024; Zhao G.-J. et al., 2025). FTO mainly targets PFKP, regulating PFKP and LDHB translation in an m6A-dependent manner to promote leukemogenesis and increasing the chromatin accessibility of glycolytic genes such as HK1 and PFKP to drive tumor development (Liu et al., 2021d; Qing et al., 2021). Several readers stabilize PFK transcripts to sustain glycolysis: IGF2BP2 stabilizes PFKL via circDHTKD1 in NSCLC (Liu Z. et al., 2024), YTHDC1 recognizes m6A sites on PFKM and LDHA to increase their mRNA stability and promote osteosarcoma growth (Mei et al., 2024), and YTHDF3 inhibits PFKL mRNA degradation to elevate PFKL expression and accelerate aerobic glycolysis and HCC progression (Zhou et al., 2022).

### 3.5 m6A and ENO1

Enolase-1 (ENO1) is a key glycolytic enzyme that catalyzes the conversion of 2-phospho-D-glycerate to phosphoenolpyruvate during aerobic glycolysis (Zhou et al., 2019). m6A regulators converge on ENO1 to activate glycolysis across several tumor types. METTL3 promotes ENO1 expression to drive glycolysis and tumorigenesis: in LUAD it cooperates with ALKBH5 and YTHDF1 to mediate ENO1 translation, and in esophageal cancer it upregulates a panel of glycolytic genes including ENO1 (SLC2A1, PFKL, PGK1, ENO1, and PKM) (Ma L. et al., 2022; Liu X.-S. et al., 2024). WTAP is another recurrent regulator of ENO1, promoting the glycolytic capacity of breast cancer cells through the ERK1/2–WTAP/ENO1 axis. The WTAP–ENO1 network serves as a therapeutic target, and mediates ENO1 m6A modification in diabetic nephropathy (Ou et al., 2021; Bai Y. et al., 2024). In addition, KIAA1429 stabilizes ENO1 mRNA in an m6A-dependent manner to promote glycolysis and ovarian cancer progression (Gan, 2023).

### 3.6 m6A and GLUT

Glucose transporters (GLUTs) are key regulatory molecules at the initial stage of glycolysis and are widely distributed in mammalian cells. They are the key transporters that control glucose transport across the cell membrane during glycolysis. Currently, 14 members of the GLUT family have been identified in the human proteome, among which abnormal overexpression of GLUT1 and GLUT3 is closely related to enhanced tumor invasiveness (Pliszka and Szablewski, 2021). m6A regulators predominantly target GLUT1 to enhance glucose uptake and aerobic glycolysis. Among the writers, METTL3 stabilizes or upregulates GLUT1 in clear cell renal cell carcinoma (ccRCC) and other tumors—directly binding the GLUT1 3′-UTR through IGF2BP2/3 and, via HDGF m6A modification, also promoting GLUT4 and ENO2 (Wang et al., 2020c; Zhan et al., 2023; Ning et al., 2024); WTAP enhances GLUT1 glycosylation and membrane transport through m6A modification of PIGT (Tan et al., 2024); KIAA1429 stabilizes GLUT1 mRNA by recognizing the m6A site of LINC00958 to promote gastric cancer glycolysis (Yang D. et al., 2021); and RBM15 upregulates GLUT1 together with HIF-1α and PFKFB3 to promote glycolysis in macrophages (Tang et al., 2024). On the reader and eraser side, circFOXK2 cooperates with IGF2BP3 to stabilize GLUT1 mRNA in OSCC (Cui et al., 2023), HNRNPC promotes glycolysis in multiple myeloma (MM) through the GLUT1/LDHA pathway (Wu and Zhu, 2024), and FTO reduces GLUT1 m6A to attenuate YTHDF2-mediated mRNA decay in liver cancer (Wang et al., 2023b), whereas hnRNPA2B1 instead reduces GLUT1 stability and translation (Hamilton et al., 1999). m6A regulators also act on other GLUT members: METTL14 promotes GLUT3 through YTHDF1 to enhance glycolysis and osteoblast differentiation (Wang Y. et al., 2025), while YTHDC1 regulates GLUT3 to instead inhibit the malignant progression and glycolysis of bladder cancer (Yan et al., 2023); for GLUT4, ALKBH5 increases GLUT4 mRNA stability in a YTHDF2-dependent manner to enhance glycolysis in breast cancer (Liu et al., 2022d).

### 3.7 m6A and LDHA

Lactate dehydrogenase (LDH) is a key enzyme in the final stage of glycolysis and is a tetrameric enzyme composed of two different subunits, LDHA and LDHB, in various combinations (Urbańska and Orzechowski, 2019). In various types of cancer cells, LDHA levels increase to adapt to anaerobic glycolysis, and LDH-A has been proposed as a biomarker for cancer diagnosis and prognosis (Petrelli et al., 2015). The expression and function of LDH are regulated by m6A methylation in a largely tumor-promoting manner, with multiple writers, erasers, and readers enhancing LDHA expression to support glycolysis. Among the writers, METTL3 increases LDHA transcription by stabilizing HIF-1α mRNA through IGF2BP3 in CRC (Zhang et al., 2022b), and RBM15 stabilizes LDHA mRNA to promote malignant behavior in LUAD (Shi et al., 2024); the erasers also act on LDHA, as ALKBH5—upon PRMT6-mediated methylation at R283—increases LDHA RNA stability to promote breast cancer (Han et al., 2024), and the R-2HG/FTO axis targets PFKP and LDHB to attenuate aerobic glycolysis in leukemia (Qing et al., 2021). On the reader side, IGF2BP1 and IGF2BP2 enhance LDHA mRNA stability to promote glycolysis in colon and prostate cancers (Zhang X.-L. et al., 2021; Jiang X. et al., 2022), YTHDC1 increases PFKM and LDHA mRNA stability to promote osteosarcoma growth (Mei et al., 2024), and hnRNPA2B1 both stabilizes LDHA mRNA in an m6A-dependent manner and, through regulation of GLUT1 and PKM2, increases LDH expression to drive pancreatic cancer proliferation (Brandi et al., 2016; Wen M. et al., 2024). In contrast, METTL14 acts as a tumor suppressor in breast cancer by reducing LDHA expression, indicating that m6A regulators can also negatively regulate LDHA (Han et al., 2024).

### 3.8 Tumor-type specificity of m6A-driven metabolic reprogramming

From the studies above, it can be further observed that m6A plays distinct roles in hematological malignancies versus solid tumors. In hematological malignancies, m6A serves as a core driver of metabolic survival, with the focus lying mainly on the hematopoietic stem cell–specific metabolic network that meets the energy demands of rapid LSC proliferation. For example, the target genes regulated by METTL3 are essential for AML (Barbieri et al., 2017); METTL14 is required for the development and maintenance of AML and for the self-renewal of leukemia stem/initiating cells (LSC/LICs), exerting its oncogenic role by regulating its mRNA targets (such as MYB and MYC) through m6A modification (Weng et al., 2018); METTL3 also promotes the translation of c-MYC, Bcl2, and PTEN mRNA in the human myeloid leukemia cell line MOLM13 (Vu et al., 2017); FTO regulates the translation of PFKP, LDHB, ASB2, and RARA mRNA in an m6A-dependent manner, promoting leukemogenesis (Qing et al., 2021); and in diffuse large B-cell lymphoma (DLBCL), WTAP increases the expression of its key target gene HK2 by elevating HK2 m6A levels, thereby promoting DLBCL (Han et al., 2021a). In solid tumors, by contrast, m6A modification serves as a critical accelerator of tumor metabolic adaptation rather than an indispensable prerequisite.

## 4 m6A and signaling pathways

The regulation of signaling pathways is inseparably linked to cancer, and their abnormal activation or inhibition is the key molecular basis for the formation of malignant phenotypes in tumors. Numerous studies have confirmed that multiple signaling pathways play a core role in tumor progression, immune regulation, metabolic reprogramming, and drug resistance. The Wnt/β-catenin pathway plays an important role in cancer progression, tumor immune suppression, and immune escape (Patel et al., 2019); the PI3K/AKT signaling pathway, as an important regulator of various biological processes, is one of the most frequently activated pathways in human malignant tumors (Hoxhaj and Manning, 2020); abnormal activation of the NF-κB pathway is often present in various malignant tumors and is closely related to tumor cell proliferation, survival, angiogenesis, invasion, metastasis, and drug resistance (Khan et al., 2020); activation of the STING signaling pathway is associated with the development of various cancers, including melanoma (Xia et al., 2016b), colorectal cancer (Xia et al., 2016a), and lung cancer (Kitajima et al., 2019); the Hippo signaling pathway is related to tumor proliferation, metastasis, cancer stem cell characteristics, and drug resistance (Cunningham and Hansen, 2022); mitogen-activated protein kinases (MAPKs) play a key role in tumor metastasis, angiogenesis, drug resistance, and tumor growth (Zhi et al., 2023); AMPK is a highly conserved serine/threonine kinase that plays a crucial role in regulating the metabolism, growth, and survival of cancer cells (Wei Q. et al., 2024); the Janus kinase/signal transducer and activator of transcription (JAK/STAT) pathway is an evolutionarily conserved signaling mode that participates in the upregulation of various proteins involved in cell proliferation, stemness, self-renewal, evasion of immune surveillance, and overall tumor progression (Sabaawy et al., 2021); hypoxia-inducible factor (HIF) consists of an unstable α subunit and a stable β subunit, and HIF-1α is widely expressed in various cells and is considered a major regulator of metabolism, cell cycle, and tumorigenesis (Silagi et al., 2021); c-Myc is an oncoprotein that sits at a key node of multiple growth-promoting signaling pathways such as ERK, PI3K, AKT, MAPK, and Wnt (Yoshida, 2018), and is closely related to tumor invasiveness, drug resistance, and poor prognosis (Mo et al., 2022); p53, as a tetrameric transcription factor, is one of the most important tumor suppressors for protecting genomic integrity and inhibiting tumorigenesis, and mutations in the p53 gene have been found in up to 50% of all cancers. Mutant p53 promotes tumor growth and progression, whereas activated p53 can reduce the activity of immunosuppressive components in the TME, such as regulatory T cells (Tregs), myeloid-derived suppressor cells (MDSCs), and programmed death-ligand 1 (PD-L1) (Alvarado-Ortiz et al., 2020), thereby alleviating tumor immune escape (Cui and Guo, 2016). The transforming growth factor β (TGF-β) pathway is indispensable for cell proliferation, differentiation, and survival, and is involved in tumor proliferation and metastasis (Peng D. et al., 2022). A large number of studies have revealed that m6A modification can participate in the pathophysiological regulation of tumors by modulating the activity of the above signaling pathways (Table 2).

**TABLE 2:** m6A regulation of signaling pathways.

m6A	Pathway	Tumor/Disease	Upstream/Downstream target	Outcome or association	References
METTL3	Wnt	TNBC	Increases FAM83D expression	Accelerates TNBC progression	Yu X. et al. (2024)
​	AKT	PDAC	Increases DDX23 expression	Promotes PDAC progression and gemcitabine resistance	Lin C. et al. (2023)
​	NF-κB	HCC	Increases ZNNT1 expression	Enhances malignant features of HCC cells	Wei H. et al. (2024)
​	STING	Radiation-induced liver disease (RILD)	Promotes TEAD1 methylation and expression	Promotes RILD progression	Wang et al. (2024c)
​	Hippo	Hepatoblastoma	Decreases LATS2 expression	Promotes hepatoblastoma progression	Zhu et al. (2024)
​	MAPK	Respiratory infection	Increases HDAC9 expression	Promotes tracheal inflammation	Zeng Y. et al. (2024)
​	AMPK	Chronic kidney disease (CKD)	Increases FOSL1 stability	Inhibits mitophagy	Liu T. et al. (2025)
​	STAT	Atherosclerosis	Increases JAK2 expression	Accelerates atherosclerosis progression	Dong G. et al. (2021)
​	HIF	Lung cancer	HIF-1α regulates METTL3 expression	Regulates lung cancer progression	Zhao Y. et al. (2025)
​	MYC	Prostate cancer	Increases SNHG7 expression	Promotes prostate cancer progression	Liu et al. (2022e)
​	p53	Prostate cancer	Enhances CircGLIS3 stability	Promotes prostate cancer development	Cheng X. et al. (2024)
​	TGF-β	Gastric cancer	Enhances Smad3 protein expression	Promotes gastric cancer proliferation and metastasis	Yuan et al. (2023)
METTL5	MAPK	Gastric cancer	Not specified	METTL5 expression negatively correlated with clinicopathological stage	Wang Z. et al. (2021)
​	MYC	HCC	Increases USP5 translation	Promotes HCC metastasis	Xia et al. (2023)
​	TGF-β	Intrahepatic cholangiocarcinoma	Mediates 18S rRNA m6A modification	Regulates intrahepatic cholangiocarcinoma development	Dai et al. (2023)
METTL14	Wnt	LUAD	Mediates upregulation of lncRNA-AC026356.1	Promotes oncogenic properties of cancer stem cells	Zhang et al. (2023c)
​	AKT	Colon cancer	Increases TMUB1 expression	Accelerates colon cancer progression and worsens overall survival	Jiang et al. (2024)
​	NF-κB	Cervical cancer	Positively regulates HOXB13 expression	Promotes cervical cancer progression	Li Q. et al. (2024)
​	STING	Ischemic stroke	Induces HDAC3 m6A modification	Reverses ischemic stroke-induced brain injury	Liang et al. (2024)
​	Hippo	TNBC	Blocks YTHDF2-mediated YAP1 transcript decay	Maintains TNBC stemness	Bai X. et al. (2024)
​	MAPK	Diabetic nephropathy	Promotes TUG1 degradation	Promotes renal tubular epithelial cell apoptosis	Zheng et al. (2023)
​	AMPK	Cervical cancer	Increases AMPK protein expression	Accelerates glycolysis	Wang et al. (2024b)
​	STAT	Thyroid cancer	Increases SOCS3 expression	Alleviates thyroid cancer progression	Zhou et al. (2024)
​	HIF	Psoriasis	METTL14 activates HIF-1α	Promotes psoriasis development	Hu Y. et al. (2023)
​	MYC	Cervical cancer	METTL14 regulates m6A level of Myc	Enhances proliferation and migration of cervical cancer cells	Hu C. et al. (2022)
​	p53	AML	Regulates MDM2 mRNA stability	Promotes AML development	Sang et al. (2022)
METTL16	Wnt	EOC	Promotes lncRNA MALAT1 degradation	Inhibits EOC development	Li C. et al. (2023)
​	AKT	Lung cancer	Regulates miR-146b m6A modification	Mediates osimertinib resistance	Sang et al. (2025)
​	NF-κB	Gastric cancer	Reduces m6A modification of UBXN1 coding sequence	Alters malignant phenotype of gastric cancer	Shi et al. (2025)
​	AMPK	Colon cancer	Increases SOGA1 expression	Promotes glycolysis	Wei W. et al. (2024)
​	HIF	HCC	HIF-1α upregulates METTL16 expression	Promotes HCC metastasis	Wang et al. (2023e)
​	MYC	Lung cancer	MYC is the upstream mediator that METTL16 regulates via transcriptional activation	Regulates lung cancer progression	Xie et al. (2024)
Hakai	HIF	Ocular disease	Hakai inhibits HIF-1α activation	Suppresses cellular metabolism and angiogenesis	Dong L. et al. (2021)
RBM15	AKT	Cervical cancer	Increases OTUB2 expression	Accelerates cervical cancer progression	Song and Wu (2023)
​	MAPK	HCC	Promotes transcriptional activation of YES1	Drives HCC progression	Cai et al. (2021)
​	HIF	Aortic aneurysm and dissection	Increases HIF-1α expression	Promotes aortic aneurysm and dissection progression	Tang et al. (2024)
​	MYC	Cervical cancer	RBM15 binds to Myc mRNA	Promotes metastasis of cervical cancer cells	Nie et al. (2023)
​	p53	Esophageal cancer	Increases KRT4 expression	Inhibits proliferation of esophageal cancer cells	Wang (2024)
​	TGF-β	TNBC	Promotes m6A methylation of TNFSF9	Mediates TNBC drug resistance	Fu et al. (2025)
VIRMA	AKT	Cholangiocarcinoma	Regulates methylation of TMED2 and PARD3B	Promotes cholangiocarcinoma proliferation and metastasis	Xu H. et al. (2023)
​	Hippo	Lymphoma	Regulates m6A modification of CHST11 mRNA	Promotes lymphoma progression	Chen X. et al. (2023)
​	MAPK	Lung cancer	Upregulates MAP3K2 expression	Promotes gefitinib resistance	Lin X. et al. (2023)
​	AMPK	Nephroblastoma	Regulates SCD m6A methylation	Promotes nephroblastoma progression	Zhu and Li (2025)
​	STAT	LUAD	Induces m6A modification of LINC01106	Enhances malignancy of LUAD cells	Xu et al. (2024)
​	HIF	Pancreatic cancer	Increases STRA6 expression	Promotes pancreatic cancer progression	Yang K. et al. (2024)
​	p53	Protein synthesis machinery studies	Triggers p53-dependent stress response	Causes forebrain developmental defects	Wu et al. (2025b)
​	TGF-β	NSCLC	Activates downstream target gene ADAR	Promotes NSCLC progression	Shan et al. (2025)
WTAP	Wnt	Endometrial cancer	Promotes GSK3β phosphorylation	Promotes cisplatin resistance	Xie W. et al. (2021)
​	AKT	ESCC	Increases PTP4A1 expression	Accelerates ESCC progression	Zou et al. (2024)
​	NF-κB	Endometrial cancer	Downregulates Cav-1 expression	Promotes endometrial cancer metastasis	Li et al. (2021d)
​	Hippo	AML	Decreases WWTR1 stability	Impairs AML cell proliferation and tumorigenesis	Hu C. et al. (2020)
​	MAPK	Colon cancer	Increases VEGFA expression	Promotes CRC development and angiogenesis	Ye et al. (2023c)
​	AMPK	HCC	Increases LKB1 expression	Inhibits HCC proliferation	Li et al. (2021a)
​	STAT	Inflammatory diseases	Not specified	WTAP/STAT3 axis closely related to inflammatory diseases	Ge et al. (2024)
​	HIF	Ovarian cancer	HIF-1α promotes WTAP expression	Promotes ovarian cancer progression	Lyu et al. (2022)
​	MYC	AML	Regulates m6A methylation of MYC mRNA	Regulates AML progression	Naren et al. (2021)
​	TGF-β	Liver fibrosis	Regulates FBLIM1 expression	Mediates liver fibrosis	Ren et al. (2025)
ZC3H13	Wnt	Cervical cancer	Increases CENPK expression	Enhances tumorigenicity and drug resistance of cervical cancer stem cells	Lin et al. (2022)
​	NF-κB	Renal cancer	Inhibits LMO2 expression	Promotes renal cancer progression	Wang H. et al. (2025)
​	MAPK	Malignant glioma	Regulates methylation of dual-specificity phosphatase 9 (DUSP9) mRNA	Induces M2 polarization of microglia	Guo et al. (2024)
​	AMPK	Cervical cancer	Increases hsa_circ_0081723 stability	Promotes cervical cancer progression	Wei Q. et al. (2024)
​	STAT	HCC	Not specified	Regulates migration and invasion of HCC cells	Wu et al. (2022)
ZCCHC4	MYC	Esophageal cancer	Regulates MYC through reactive oxygen species (ROS)	Mediates cisplatin sensitivity in esophageal cancer	Yao et al. (2025)
FTO	Wnt	HNSCC	Increases CTNNB1 expression	Accelerates tumor progression	Zhang Y. et al. (2021)
​	AKT	Gastric cancer	Increases circFAM192A stability	Promotes gastric cancer cell proliferation	Wu X. et al. (2024)
​	NF-κB	Lung injury	Inhibits miR-192 production	Increases M1 macrophage polarization and activates AKT/NF-κB inflammatory pathway	Wu S. et al. (2024)
​	STING	Rheumatoid arthritis	Participates in TNF-α-mediated inflammatory response via CMPK2	Increases proliferation and migration of RA cells	Jin et al. (2024)
​	Hippo	Cervical cancer	Increases BMP4 expression	Promotes cervical cancer progression	Huang et al. (2023)
​	MAPK	Gastric cancer	Increases MOXD1 expression	Associated with poor prognosis in gastric cancer	Lai et al. (2024)
​	AMPK	NSCLC	Destabilizes SESN2 mRNA	Promotes malignant progression of NSCLC	Wang et al. (2024e)
​	​	Gastric cancer	Increases PRKAA1 expression	Promotes glycolysis	Zhang et al. (2022c)
​	STAT	Bladder cancer	Increases STAT3 expression	Accelerates bladder cancer progression	Sun et al. (2023)
​	HIF	Esophageal cancer	Enhances LINK-A stability	Promotes esophageal cancer progression	Nan et al. (2023)
​	MYC	CRC	Increases MYC expression	Promotes CRC progression	Yue et al. (2020)
​	​	Cervical cancer	Regulates m6A modification of Myc transcripts	Regulates cervical cancer progression	Zou et al. (2019)
​	p53	Gastric cancer	Regulates specificity protein 1 and Aurora kinase B	Promotes gastric cancer progression	Zeng X. et al. (2024)
​	TGF-β	HCC	Regulates m6A modification of BUB1 mRNA	Mediates HCC metastasis	Zhang et al. (2025b)
ALKBH5	Wnt	Glioma	Destabilizes FOXO1 and reduces its expression	Promotes tumor development	Wang C. et al. (2025)
​	AKT	HCC	Downregulates m6A methylation of TMCO3 mRNA	Inhibits HCC progression	Li et al. (2025)
​	NF-κB	HCC	Regulates m6A demethylation of TIRAP mRNA	Improves HCC radiosensitivity	Chen Y. et al. (2023)
​	STING	RILD	Binds to HMGB1	Reduces liver inflammation	Chen G. et al. (2021)
​	Hippo	Colon cancer	Reduces m6A modification of circXPO1	Inhibits colon cancer cell growth and liver metastasis	Zhu and Zhang (2024)
​	MAPK	MM	Enhances TRAF1 mRNA stability	Promotes MM cell growth	Qu et al. (2022)
​	AMPK	GBM	Promotes PYCR2 expression	Promotes GBM cell proliferation, migration, and invasion	Li L. et al. (2023)
​	STAT	Osteosarcoma	Increases SOCS3 expression	Inhibits tumor cell proliferation and cell cycle	Yang Z. et al. (2022)
​	HIF	Breast cancer	Increases NANOG expression	Promotes breast cancer development	Zhang et al. (2016)
​	MYC	Esophageal cancer	Regulates m6A modification and stability of c-Myc mRNA	Regulates esophageal cancer progression	Qiao et al. (2023)
YTHDF1	Wnt	HCC	Upregulates SLC2A1-DT expression	Enhances glycolysis	Zeng Z. et al. (2024)
​	AKT	Breast cancer	Increases GPRC5A translation	Accelerates breast cancer progression	Ou et al. (2024)
​	NF-κB	Medication-induced headache (MIH)	Enhances TRAF6 protein expression	Promotes NF-κB activation	Ouyang et al. (2022)
​	STING	Melanoma	Not specified	Improves efficacy of radioimmunotherapy	Wen C. et al. (2024)
​	MAPK	Inflammatory bone disease	Decreases Tnfrsf11a mRNA stability	Suppresses inflammatory osteoclast differentiation and bone resorption	He M. et al. (2022)
​	AMPK	Lipid metabolism studies	Inhibits AMPK signaling pathway	Causes lipid metabolism disorders	Chen S. et al. (2023)
​	STAT	Embryonic stem cell studies	Promotes STAT3 protein	Maintains self-renewal and pluripotency of piPSCs	Wu et al. (2019)
​	HIF	Lung cancer	Regulates methylation and expression of HIF genes	Promotes hypoxia-related tumor progression	Shi Y. et al. (2019)
​	MYC	OSCC	Enhances c-Myc stability	Promotes tumor cell progression	Han et al. (2021b)
​	p53	Acute cerebral ischemia	Drives p53 protein translation	Promotes oxidative stress and ferroptosis	Chang et al. (2025)
​	TGF-β	Prostate cancer	Regulates m6A methylation of GDF15	Mediates prostate cancer progression	Jiang et al. (2026)
YTHDF2	Wnt	GBM	Increases degradation of APC and GSK3β mRNA	Accelerates GBM progression	Yu P. et al. (2024)
​	AKT	GBM	Decreases stability of EPHB3 and TNFAIP3	Promotes tumor development	Chen et al. (2022d)
​	NF-κB	Glioma	Promotes UBXN1 mRNA degradation	Promotes glioma progression	Chai et al. (2021)
​	STING	HCC	STING pathway upregulates YTHDF2 expression	Enhances outcomes of immunotherapy intervention	Yang Z. et al. (2024)
​	Hippo	Lymphoma	Decreases CHST11 expression	Leads to Hippo-YAP signaling inactivation	Chen X. et al. (2023)
​	MAPK	Inflammatory bone disease	Increases MAP2K4 expression	Exacerbates inflammatory response	Fang et al. (2021)
​	AMPK	Skeletal muscle development studies	Decreases serine/threonine kinase 11 (STK11) expression	Inhibits myoblast proliferation and myogenic differentiation	Deng et al. (2024)
​	STAT	Gastrointestinal tumors	Degrades IL11 mRNA	Inhibits STAT3 phosphorylation and tumor growth	Hou et al. (2019)
​	HIF	HCC	Hypoxia suppresses METTL14 in a HIF-1α-dependent manner	Eliminates ferroptosis in HCC cells	Fan et al. (2021)
​	MYC	Glioma	Increases MYC stability	Promotes glioma progression	Dixit et al. (2021)
​	p53	CRC	Increases TYMS mRNA stability	Promotes CRC cell proliferation	Zhou et al. (2025)
YTHDF3	Wnt	HCC	Inhibits NKD1 transcription and translation	Promotes HCC cell invasion and metastasis	Chen et al. (2024b)
​	AKT	Cervical cancer	Catalyzes m6A modification of PDE3A mRNA	Inhibits cervical cancer progression	Liu Y. et al. (2024)
​	Hippo	Papillary thyroid carcinoma	Decreases P4HA2 mRNA stability	Promotes papillary thyroid carcinoma development	Ding et al. (2025)
​	STAT	HCC	Increases EGFR expression	Accelerates HCC progression	Hu B. et al. (2023)
​	MYC	Pancreatic cancer	YTHDF3 recognizes m6A modification in MYC mRNA	Promotes pancreatic cancer progression	Zhang H. et al. (2024)
​	p53	Uveal melanoma (UVM)	TRIM2 is an m6A modification substrate of YTHDF3	Regulates UVM progression	Li et al. (2026)
YTHDC1	Wnt	Glioma	Destabilizes FOXO1	Accelerates glioma progression	Wang C. et al. (2025)
​	AKT	Bladder cancer	Decreases PTEN expression	Promotes cisplatin resistance	Su et al. (2023)
​	NF-κB	Placental dysfunction	Decreases circMPP1 expression	Inhibits NF-κB and MAPK3 signaling pathways	Wang et al. (2023a)
​	MAPK	Renal cancer	Downregulates ANXA1/MAPK pathway	Associated with poor prognosis	Li W. et al. (2022)
​	STAT	Ovarian cancer	Increases PIK3R1 expression	Regulates ovarian cancer development	Wang et al. (2023d)
​	HIF	Pancreatic cancer	Increases miR-30 d expression	Inhibits PDAC tumorigenesis	Hou et al. (2021)
​	MYC	HCC	Transcriptional activation of MYC	Promotes HCC development	Tan et al. (2023)
​	p53	DNA damage studies	YTHDC1 is a major regulator of p53 expression	Regulates DNA damage response	Elvira-Blázquez et al. (2024)
​	TGF-β	TNBC	Promotes SMAD3 expression	Promotes TNBC metastasis	Tan et al. (2022)
YTHDC2	AKT	Nasopharyngeal carcinoma	Increases IGF1R protein levels	Promotes radioresistance of nasopharyngeal carcinoma cells	He et al. (2020)
​	NF-κB	Lung cancer	Not specified	Inhibits proliferation and migration of lung cancer cells	Wang J. et al. (2021)
​	MAPK	Colon cancer	Not specified	Promotes CRC cell apoptosis	Yu et al. (2020)
​	STAT	Esophageal cancer	Not specified	Regulates esophageal cancer progression	Yang N. et al. (2020)
​	HIF	Colon cancer	YTHDC2 promotes HIF-1α translation	Promotes metastasis of colon tumor cells	Tanabe et al. (2016)
NKAP	AKT	Pancreatic cancer	Promotes maturation of miR-25-3p	Accelerates tumor progression	Zhang et al. (2019)
IGF2BP	Wnt	Breast cancer	Recognizes m6A methylation of DROSHA	Accelerates breast cancer progression	Tabnak et al. (2023)
​	AKT	Prostate cancer	Promotes VEGFA expression	Promotes prostate cancer cell proliferation and migration	Chen et al. (2024d)
​	NF-κB	Hepatic failure	Decreases RORα expression	Increases liver injury and inflammation	Cheng et al. (2024a)
​	STING	RILD	Enhances TEAD1 mRNA stability	Promotes RILD	Wang et al. (2024c)
​	Hippo	Bladder cancer	MNX1-AS1 is transcriptionally activated by TEAD4	Promotes bladder cancer tumorigenesis, progression, and metastasis	Liu et al. (2022f)
​	MAPK	ESCC	Recognizes and binds MKK6 and MAPK14 mRNA	Promotes ESCC metastasis and proliferation	Zhao et al. (2023)
​	AMPK	CKD	Attenuates FOSL1 m6A modification	Attenuates glomerular injury	Liu T. et al. (2025)
​	STAT	NSCLC	Increases XBP1 expression	Promotes proliferation, migration, and invasion of NSCLC cells	Liang et al. (2025)
​	HIF	Gastric cancer	Directly binds to the coding region of HIF1A mRNA	Promotes gastric cancer progression	Jiang L. et al. (2021)
​	MYC	Bladder cancer	Regulates MYC expression	Mediates bladder cancer progression	Xie F. et al. (2021)
​	​	HCC	Regulates Myc mRNA stability	Mediates HCC progression	Wei L. et al. (2021)
​	p53	HCC	Regulates FBXO43 stability	Mediates HCC progression	Zhou H. et al. (2023)
​	TGF-β	Pancreatic cancer	Regulates EMP1 mRNA stability	Regulates pancreatic cancer microenvironment remodeling	Liu L. et al. (2025)
HNRNPC	Wnt	Glioma	Interacts with DDX11-AS1	Accelerates glioma progression	Xiang et al. (2022)
​	AKT	CLL	Increases CPT1A expression	Accelerates proliferation of CLL cells	Wu Z. et al. (2023)
​	MAPK	Gastric cancer	Regulates MNK2 pre-mRNA	Regulates gastric cancer progression	He Q. et al. (2022)
​	STAT	Lung cancer	Interacts with KH-type splicing regulatory protein	Accelerates lung cancer progression	Yan et al. (2019)
​	HIF	HCC	HNRNPC promotes HIF-1α expression	Promotes HCC invasiveness and metastasis	Liu et al. (2022b)
​	MYC	HCC	Increases Myc mRNA stability	Promotes HCC metastasis	Duan et al. (2024)
​	TGF-β	Scar studies	Increases WDR77 stability	Promotes scar formation	Zhan et al. (2026)
HNRNPA2B1	Wnt	CRC	Regulates TCF7L2 mRNA stability	Promotes cetuximab resistance and tumor metastasis	Liu et al. (2022c)
​	AKT	NSCLC	Increases MEG3 expression	Promotes NSCLC cell proliferation and invasion	Li C. et al. (2023)
​	NF-κB	Pneumonia	Promotes sorting of miR-103-3p into EPC-Exos	Suppresses pulmonary inflammation	Yang et al. (2025)
​	MAPK	Gastric cancer	Increases CENPF expression	Promotes gastric cancer metastasis	Xu P. et al. (2023)
​	STAT	Breast cancer	Not specified	Accelerates breast cancer progression	Gao et al. (2021)
​	HIF	Basic tumor research	hnRNPA2B1 binds to HIF-1α and promotes its expression	Promotes tumor progression	Soung et al. (2019)
​	MYC	Renal cancer	Myc binds to hnRNPA2B1 promoter	Promotes renal cancer progression	Liu et al. (2020)
EIF3	Wnt	Intrahepatic cholangiocarcinoma	Increases CCND1 expression	Accelerates intrahepatic cholangiocarcinoma cell proliferation and migration	Wei et al. (2022)
​	AKT	Prostate cancer	Not specified	Accelerates prostate cancer progression	Hu et al. (2019)
​	NF-κB	*Vibrio* harveyi immune evasion	Increases K27-linked ubiquitination of MyD88	Negatively regulates NF-κB pathway	Chen et al. (2022c)
​	Hippo	Breast cancer	Regulates YAP deubiquitination	Promotes invasion and migration of breast cancer cells	Zhou et al. (2020)
​	MAPK	Laryngeal squamous cell carcinoma (LSCC)	Increases MAP2K2 expression	Promotes LSCC progression	Tan J. et al. (2025)
​	AMPK	eIF3a-tumor studies	Controls synthesis of small GTPase Rheb	Activates AMPK signaling pathway	Ma S. et al. (2022)
​	STAT	Lung cancer	Not specified	Accelerates lung cancer metastasis	Esteves et al. (2020)
​	HIF	HCC	Not specified	Promotes HCC progression	Miao et al. (2019)
​	MYC	CRC	Increases MYC stability	Promotes tumor cell growth and tumorigenicity	Pan et al. (2023)

AML, acute myeloid leukemia; CKD, chronic kidney disease; CLL, chronic lymphocytic leukemia; CRC, colorectal cancer; EOC, epithelial ovarian cancer; ESCC, esophageal squamous cell carcinoma; GBM, glioblastoma; HCC, hepatocellular carcinoma; HNSCC, head and neck squamous cell carcinoma; LSCC, laryngeal squamous cell carcinoma; LUAD, lung adenocarcinoma; MIH, medication-induced headache; MM, multiple myeloma; NSCLC, non-small cell lung cancer; OSCC, oral squamous cell carcinoma; PDAC, pancreatic ductal adenocarcinoma; RILD, radiation-induced liver disease; TNBC, triple-negative breast cancer; UVM, uveal melanoma.

### 4.1 m6A and Wnt

Among m6A methyltransferases, most family members facilitate tumor progression by activating the Wnt/β-catenin pathway. In TNBC, METTL3 positively regulates the expression of FAM83D, thereby activating the Wnt/β-catenin pathway (Yu X. et al., 2024); METTL14 can work in synergy with IGF2BP2 to mediate the upregulation of lncRNA-AC026356.1, activating the Wnt signaling pathway and promoting the oncogenic properties of cancer stem cells in LUAD (Zhang et al., 2023c); By contrast, METTL16 can inhibit the development of epithelial ovarian cancer (EOC) by binding and promoting the degradation of lncRNA MALAT1, thereby downregulating the expression of β-catenin (Li C. et al., 2023). In endometrial cancer, WTAP activates the Wnt/β-catenin pathway and confers cisplatin resistance in tumor cells (Xie W. et al., 2021); ZC3H13-mediated m6A methylation of CENPK can activate Wnt signaling (Lin et al., 2022). The two canonical m6A demethylases FTO and ALKBH5 universally activate Wnt/β-catenin signaling to promote tumor growth. In head and neck squamous cell carcinoma, FTO increases the expression of CTNNB1, the gene encoding β-catenin, thereby accelerating tumor progression (Zhang Y. et al., 2021); in glioma, ALKBH5 destabilizes FOXO1 and reduces its expression through m6A demethylation in a YTHDC1-dependent manner, promoting Wnt/β-catenin signal transduction (Wang C. et al., 2025). For m6A reader proteins, YTHDF2 and YTHDF3 promote Wnt signaling by eliminating pathway repressors. In GBM, YTHDF2 promotes the degradation of APC and GSK3β mRNAs, which are negative regulators of the Wnt/β-catenin pathway, accelerating GBM progression (Yu P. et al., 2024); YTHDF3 inhibits the transcription and translation of NKD1, activating the Wnt/β-catenin pathway and promoting the invasion and metastasis of HCC cells (Chen et al., 2024b); it has been shown that m6A methylation of DROSHA and its recognition by IGF2BP2 can enable DROSHA to interact with β-catenin, promoting the progression of breast cancer (Tabnak et al., 2023); DDX11-AS1 can interact with HNRNPC to activate the Wnt/β-catenin and AKT pathways, promoting the tumorigenesis and progression of glioma (Xiang et al., 2022); MIR100HG and hnRNPA2B1 interact to regulate the stability of TCF7L2 mRNA, controlling the transcriptional activity of Wnt signaling in colorectal cancer and affecting cetuximab resistance and tumor metastasis (Liu et al., 2022c); EIF3H stabilizes CCND1 expression during tumorigenesis, and CCND1 promotes the proliferation and migration of intrahepatic cholangiocarcinoma cells through the Wnt/β-catenin signaling pathway (Wei et al., 2022).

### 4.2 m6A and AKT

As the primary m6A methyltransferases, METTL3 and METTL14 serve as pivotal upstream activators of AKT signaling in multiple cancers.METTL3 regulates DDX23 mRNA m6A methylation to promote the activation of the PI3K/Akt signaling pathway, thereby facilitating the progression of PDAC (Lin C. et al., 2023). In addition, in studies on renal cancer (Li X. et al., 2017), ovarian cancer (Bi et al., 2021), esophageal cancer (Xia et al., 2020), lung cancer (Cheng et al., 2022), bladder cancer (Zhu et al., 2023), and gastric cancer (Lin et al., 2019), METTL3 has been shown to regulate the mTOR-AKT signaling pathway and participate in the malignant progression of tumors; in colon cancer, METTL14 and YTHDF2 can mediate the regulation of TMUB1, which activates the AKT signaling pathway by interacting with the autocrine motility factor receptor (AMFR), thereby promoting tumor progression (Jiang et al., 2024). Meanwhile, METTL14 has been confirmed to regulate the mTOR-AKT signaling pathway in renal cancer (Zhang C. et al., 2024), endometrial cancer (Liu et al., 2018), pancreatic cancer (Ma et al., 2024), esophageal cancer (Liu et al., 2021f), gastric cancer (Liu et al., 2021c), cervical cancer (Li L. et al., 2024), neuroblastoma (Wang et al., 2024d), and liver cancer (Shi et al., 2020). In lung cancer, METTL16 is responsible for miR-146b m6A modification, activating PI3K/AKT to promote osimertinib resistance (Sang et al., 2025); WTAP regulates the expression of PTP4A1 by mediating m6A modification, activating the AKT-mTOR pathway and promoting the proliferation of esophageal squamous cell carcinoma (ESCC) cells (Zou et al., 2024); furthermore, in pancreatic cancer (Li B.-Q. et al., 2019), ovarian cancer (Yu et al., 2019), and tongue squamous cell carcinoma (Liu F. et al., 2025), WTAP has also been confirmed to activate the PI3K/AKT pathway; Song et al. (Song and Wu, 2023) demonstrated that overexpression of RBM15 increases the m6A methylation level of OTUB2 in cervical cancer cells, thereby activating the AKT/mTOR signaling pathway and promoting the malignant behavior of cervical cancer cells; VIRMA mediates the methylation of TMED2 and PARD3B, subsequently activating the Akt/GSK/β-catenin signaling pathway, promoting the proliferation and metastasis of cholangiocarcinoma (Xu H. et al., 2023). On the eraser side, FTO and ALKBH5 keep AKT/mTOR signaling active in a range of tumors. Studies have shown that FTO binds to circFAM192A in an m6A-dependent manner to activate the mTOR signaling pathway and promote the proliferation of gastric cancer cells (Wu X. et al., 2024); in liver cancer, ALKBH5 downregulates the m6A methylation level of TMCO3 mRNA and thereby regulates the AKT signaling pathway (Li et al., 2025); furthermore, ALKBH5 also regulates the mTOR-AKT signaling pathway in pancreatic cancer (He et al., 2021), gastric cancer (Fang et al., 2022). The m6A reader proteins exhibit diverse context-dependent functions in tuning AKT signaling across distinct tumor types. In breast cancer, the m6A methylation of GPRC5A mRNA is regulated by METTL3 and YTHDF1, thereby regulating the mTORC1/p70S6K signaling pathway (Ou et al., 2024); in GBM, YTHDF2 binds to sites on EPHB3 and TNFAIP3, thereby activating the PI3K/Akt and NF-κB signaling (Chen et al., 2022d); furthermore, in prostate cancer (Li J. et al., 2020) and lymphoma (Chen et al., 2024c), YTHDF2 can also regulate the mTOR/AKT signaling pathway; METTL3 catalyzes the m6A modification of PDE3A mRNA through YTHDF3, thereby regulating the AKT/mTOR signaling pathway to inhibit the progression of cervical cancer (Liu Y. et al., 2024); in bladder cancer, YTHDC1 regulates the PTEN/PI3K/AKT signaling pathway in an m6A-dependent manner to affect the efficacy of chemotherapy (Su et al., 2023); in nasopharyngeal carcinoma, the upregulation of YTHDC2 increases the level of IGF1R protein, thereby activating the PI3K-AKT/S6 signaling pathway (He et al., 2020); furthermore, CDK12 inhibits IGFBP3 involved in the AKT signaling pathway, promoting VEGFA expression and driving the proliferation and angiogenesis of prostate cancer cells (Chen et al., 2024d); IGF2BPs play a regulatory role in the AKT signaling pathway in colon cancer (Liu et al., 2022g), breast cancer (Jiang T. et al., 2022), and endometrial cancer (Ruan et al., 2023). In chronic lymphocytic leukemia (CLL), circTET2 interacts with HNRNPC to activate the PI3K-AKT-mTORC1 signaling pathway, participating in the lipid metabolism and proliferation of CLL cells (Wu Z. et al., 2023); in NSCLC, inhibition of HNRNPA2B1 reduces MEG3 m6A levels, and MEG3 can upregulate PTEN and inactivate PI3K/AKT signaling (Li K. et al., 2023); in HCC, the interaction of overexpressed eIF3i with AKT1 prevents the activation of AKT1 signaling (Bracic Tomazic et al., 2021); eIF3c exerts an oncogenic effect in prostate cancer by regulating PI3K/Akt/NF-κB signaling (Hu et al., 2019).

### 4.3 m6A and NF-κB

The m6A methyltransferases serve as essential upstream regulators of the NF-κB signaling cascade, exerting widespread regulatory effects on tumor malignant progression and inflammatory responses across multiple human diseases. METTL3 and METTL16 can enhance the stability of ZNNT1 transcripts by mediating m6A modification, thereby stimulating AGER/NF-κB signaling and enhancing the malignant characteristics of HCC cells (Wei H. et al., 2024); moreover, METTL3 has been confirmed to regulate the NF-κB signaling pathway in glioma (Chang et al., 2021), bladder cancer (Cheng et al., 2019), lung cancer (Jin et al., 2021), and pancreatic cancer (Wang et al., 2023c). METTL14 plays a crucial role in macrophage inflammation in atherosclerosis through the NF-κB/IL-6 signaling pathway (Zheng et al., 2022); in studies on cervical cancer (Li Q. et al., 2024) and liver injury and fibrosis (Wang et al., 2024g), the regulatory relationship between METTL14 and the NF-κB signaling pathway has also been discovered. In gastric cancer, METTL16 reduces the m6A modification level of the UBXN1 coding sequence, thereby regulating the NF-κB pathway and altering the malignant phenotype of gastric cancer (Shi et al., 2025); in endometrial cancer, WTAP can downregulate the expression of Cav-1, activate the NF-κB signaling pathway, and promote tumor progression (Li et al., 2021d); ZC3H13 can inhibit LMO2 expression, and LMO2 regulates the progression of ccRCC through the GATA2-BEX1-NF-κB signaling cascade (Wang H. et al., 2025); Canonical m6A demethylases FTO and ALKBH5 are critically involved in NF-κB-mediated inflammatory regulation and tumor radiosensitivity. It has been found that FTO overexpression can reduce the m6A modification of pri-miR-192, thereby regulating M1 macrophage polarization and the activation of the AKT/NF-κB inflammatory pathway (Wu S. et al., 2024); in HCC, radiation-induced ALKBH5 mediates the m6A demethylation of toll/interleukin-1 receptor domain-containing adaptor protein (TIRAP) mRNA and activates its downstream NF-κB pathway, improving the radiosensitivity of HCC (Chen Y. et al., 2023). Multiple m6A reader proteins act as pivotal modulators of NF-κB signaling, regulating inflammatory cytokine secretion and tumor progression through diverse m6A-dependent mechanisms. Ouyang et al. (Ouyang et al., 2022) demonstrated that YTHDF1 can enhance TRAF6 protein expression, promoting the expression of inflammation-related factors IL-6 and NF-κB; YTHDF2 can promote the degradation of UBXN1, thereby affecting the NF-κB signaling pathway and promoting glioma progression (Chai et al., 2021); YTHDC1 reduces the expression of circMPP1 through m6A modification, activating the NF-κB and MAPK3 signaling pathways (Wang et al., 2023a); IGF2BP3 can mediate the production of pro-inflammatory cytokines through regulation of the NF-κB signaling pathway (Cheng K. et al., 2024); hnRNPA2B1 promotes the sorting of miR-103-3p into EPC-Exos and regulates the NF-κB pathway (Yang et al., 2025); *Vibrio* harveyi-induced eIF3k expression can enhance the K27-linked ubiquitination of MyD88 mediated by the E3 ligase Nrdp1, activating the NF-κB pathway (Chen et al., 2022c).

### 4.4 m6A and STING

In general, m6A methyltransferases and demethylases primarily facilitate cGAS-STING pathway activation to trigger immune and inflammatory responses, whereas m6A reader proteins mostly serve as negative regulators to restrict excessive STING signaling. METTL3, as a core regulatory factor, can promote the m6A methylation and expression of TEAD1, which is then recognized by IGF2BP2 to activate the STING pathway (Wang et al., 2024c); YTHDF2 can recognize the m6A sites on METTL3-modified STING mRNA and promote the degradation of STING mRNA, thereby negatively regulating the STING signaling pathway (Geng et al., 2023); METTL14 induces the m6A modification of HDAC3 in an IGF2BP3-dependent manner and activates the cGAS-STING pathway (Liang et al., 2024); FTO and ALKBH5 are key modulators of STING-mediated inflammation and immune regulation. Studies have confirmed that the FTO-CMPK2 pathway plays a crucial role in regulating synovial inflammation by mediating the cGAS/STING pathway (Jin et al., 2024); ALKBH5 binds to m6A-modified HMGB1 and subsequently regulates STING signaling activation (Chen G. et al., 2021); research has shown that the STING/IFN-1 signaling induced by ionizing radiation can upregulate YTHDF1 expression, and the elevated YTHDF1 in turn triggers STING degradation by increasing lysosomal cathepsin levels (Wen C. et al., 2024); in liver cancer, oxaliplatin upregulates YTHDF2 expression by activating the STING signaling pathway (Yang Z. et al., 2024).

### 4.5 m6A and Hippo

METTL3, METTL14, and WTAP are the principal methyltransferases governing Hippo signaling in a range of malignancies. In hepatoblastoma, METTL3 was identified as a key component for LATS2 mRNA m6A modification at specific sites in the 5′UTR; YTHDF2 can recognize the m6A modification sites, resulting in decreased LATS2 expression and inhibiting ferroptosis through the YAP1/ATF4/PSAT1 axis, thereby promoting tumor progression (Zhu et al., 2024). In colon cancer (Li Y. et al., 2022) and breast cancer (Xu Y. et al., 2023), a regulatory role of METTL3 on the Hippo signaling pathway has also been suggested. METTL14 can increase the expression of YAP1 by blocking YTHDF2-mediated transcript decay, thereby activating Hippo signaling (Bai X. et al., 2024); miR-550-1 targets WTAP to regulate m6A levels and modulate the Hippo signaling pathway, thereby regulating AML cell proliferation and tumorigenesis (Hu C. et al., 2020); in lymphoma, YTHDF2 can bind to KIAA1429-mediated m6A modification of CHST11 mRNA, thereby regulating the Hippo-YAP pathway (Chen X. et al., 2023); FTO and ALKBH5 adjust Hippo signaling in a tumor-specific manner to shape malignant progression. Studies have shown that FTO can promote cervical cancer progression by regulating the BMP4/Hippo/YAP1/TAZ pathway *in vitro* and *in vivo* (Huang et al., 2023); in colon cancer, downregulation of ALKBH5 enhances the IGF2BP2-mediated m6A modification of circXPO1, thereby activating the Hippo-YAP pathway (Zhu and Zhang, 2024). Multiple m6A reader proteins are indispensable for the fine regulation of Hippo signaling, participating in tumor metabolism and malignant progression through diverse molecular mechanisms. In papillary thyroid carcinoma, YTHDF3 recognizes P4HA2 m6A modification and reduces the stability of P4HA2 mRNA, regulating tumor glycolytic metabolism through SAV1/YAP1/Hippo signaling (Ding et al., 2025); the MNX1AS1/IGF2BP3 axis inhibits the Hippo signaling pathway and affects bladder cancer tumorigenesis and progression (Liu et al., 2022f); EIF3H can deubiquitinate YAP, thereby regulating Hippo-YAP signaling and promoting the invasion and migration of breast cancer cells (Zhou et al., 2020).

### 4.6 m6A and MAPK

The core methyltransferases act upstream to activate MAPK signaling, driving tumor progression, inflammation, and angiogenesis through distinct m6A-dependent routes. Studies have shown that the METTL3/IGF2BP3 axis increases the expression of HDAC9, thereby promoting the activation of the MAPK signaling pathway (Zeng Y. et al., 2024); METTL3 reduces oxidative stress and inflammatory damage caused by *Staphylococcus aureus* infection through the MAPK/NF-κB/JAK2-STAT3 pathway (Wu et al., 2025a); Wang et al. (Wang Z. et al., 2021) demonstrated that GSEA bioinformatics analysis revealed that genes in the METTL5 low-expression group were enriched in several oncogenic signaling pathways, such as MAPK, JAK-STAT, Wnt, and mTOR; METTL14 activates the MAPK1/ERK signaling pathway by mediating the m6A modification of TUG1, promoting renal tubular epithelial cell apoptosis (Zheng et al., 2023); in colon cancer, WTAP regulates VEGFA, which is recognized by YTHDC1 and activates the MAPK signaling pathway, jointly promoting the development and angiogenesis of CRC (Ye et al., 2023c); in HCC, RBM15-mediated m6A modification in an IGF2BP1-dependent manner contributes to the transcriptional activation of YES1, thereby activating the MAPK pathway and promoting tumor progression (Cai et al., 2021). Studies have shown that the miR-200c-3p/ZC3H13/DUSP9/p-ERK pathway can induce M2 polarization, demonstrating that ZC3H13 can regulate the MAPK signaling pathway (Guo et al., 2024); in lung cancer, KIAA1429 can upregulate the expression of MAP3K2, activate the JNK/MAPK pathway, and promote drug resistance (Lin X. et al., 2023); FTO and ALKBH5 promote MAPK pathway activation to drive malignant progression. In gastric cancer, FTO targets MOXD1 mRNA and promotes its expression, thereby activating the MAPK signaling pathway (Lai et al., 2024); ALKBH5 can enhance the stability of TRAF1 mRNA to regulate TRAF1 expression, thereby activating the NF-κB and MAPK signaling pathways and promoting the growth of MM cells (Qu et al., 2022); The m6A reader proteins exert bidirectional regulatory effects on MAPK signaling, exhibiting significant tumor-specific functional heterogeneity and participating in inflammation modulation and tumor phenotypic transformation, studies have shown that deletion of YTHDF1 can inhibit the phosphorylation levels of key proteins in the NF-κB, MAPK, and PI3K-AKT signaling pathways (He M. et al., 2022); silencing of YTHDF2 can increase MAP4K4 mRNA expression, thereby activating the MAPK and NF-κB signaling cascades and exacerbating the inflammatory response (Fang et al., 2021); YTHDC1 can inhibit the progression of renal cancer cells by downregulating the ANXA1/MAPK pathway (Li W. et al., 2022); overexpression of YTHDC2 can regulate the p38 MAPK signaling pathway to promote CRC cell apoptosis and improve the prognosis of CRC patients (Yu et al., 2020); in esophageal cancer, PS15 interacts with IGF2BP1 to promote the translation of core p38 MAPK pathway proteins, regulating ESCC metastasis and proliferation (Zhao et al., 2023); in gastric cancer, LINC00924 can regulate MNK2 pre-mRNA by binding to hnRNPC, thereby regulating the p38 MAPK/PPARα signaling pathway (He Q. et al., 2022); in pancreatic cancer, hnRNPA2B1 interacts with linc01232 to activate the MAPK/ERK signaling pathway, accelerating tumor metastasis (Meng et al., 2020); in gastric cancer, HNRNPA2B1 can stabilize CENPF mRNA and activate the MAPK signaling pathway, thereby promoting tumor metastasis (Xu P. et al., 2023); EIF3B activates the ERK/MAPK pathway by stabilizing MAP2K2, promoting the progression of laryngeal squamous cell carcinoma (Tan J. et al., 2025).

### 4.7 m6A and AMPK

Several methyltransferases sit upstream of AMPK signaling, shaping its activation and tumor metabolic progression through distinct mechanisms. METTL3 can regulate the AMPK/mTOR signaling pathway by mediating the m6A modification of FOSL1 in an IGF2BP2-dependent manner (Liu T. et al., 2025); in cervical cancer, abnormal expression of METTL14 can regulate the m6A RNA methylation and protein expression of AMPK, thereby accelerating glycolysis (Wang et al., 2024b); in colon cancer, METTL16 binds to IGF2BP1 to enhance SOGA1 expression and mRNA stability, promoting the ubiquitination of the AMPK complex (Wei W. et al., 2024); WTAP can increase the stability of LKB1 mRNA, thereby activating AMPK signaling (Li et al., 2021a); in cervical cancer, ZC3H13 can regulate the AMPK/p53 signaling pathway (Wei Q. et al., 2024); VIRMA can mediate SCD m6A methylation and thereby promote the progression of nephroblastoma through the AMPK pathway (Zhu and Li, 2025). Canonical m6A demethylases participate in AMPK-mTOR signaling modulation and form reciprocal regulatory loops with the AMPK cascade. FTO regulates the AMPK-mTOR signaling pathway to destabilize SESN2 mRNA and promote the malignant progression of NSCLC (Wang et al., 2024e); Li L. et al. (2023) demonstrated that PYCR2 can enhance the expression of ALKBH5 through the AMPK/mTOR pathway. The readers regulate AMPK signaling in both directions, thereby influencing lipid metabolism, proliferation, and differentiation. With YTHDF1 knockdown upstream of miR-27b, TCS causes lipid metabolism disorders by inhibiting the AMPK signaling pathway (Chen S. et al., 2023); YTHDF2 deletion can inhibit myoblast proliferation and myogenic differentiation by activating the AMPK signaling pathway (Deng et al., 2024); Ma S. et al. (2022) proved that eIF3a can regulate AMPK activation by controlling the synthesis of the small GTPase Rheb.

### 4.8 m6A and STAT

For JAK-STAT signaling, the methyltransferases act upstream but work in both directions, promoting or suppressing tumors depending on the disease context. Studies have confirmed that METTL3 can regulate the JAK2/STAT3 pathway through IGF2BP1 to mediate the progression of atherosclerosis (Dong G. et al., 2021); in thyroid cancer, METTL14 can increase SOCS3 expression and alleviate tumor progression by inhibiting the JAK2/STAT3 pathway (Zhou et al., 2024); METTL14 induces m6A methylation of TRIM27, which is recognized by IGF2BP2 and activates the STAT3 signaling pathway (Chen et al., 2024e). Ge et al. (Ge et al., 2024) showed that the NF-κB/WTAP/STAT3 axis is closely related to inflammatory diseases, and WTAP is an ideal therapeutic target for many inflammatory diseases and cancers; Wu et al. (Wu et al., 2022) confirmed that ZC3H13 may be involved in transcriptional dysregulation or the regulation of the JAK-STAT signaling pathway in cancer; KIAA1429 induces the m6A modification of LINC01106 through the JAK/STAT3 pathway to enhance the malignancy of LUAD cells (Xu et al., 2024); FTO and ALKBH5 adjust JAK-STAT activity through separate m6A-dependent routes, with markedly tumor-specific outcomes. In bladder cancer, FTO enhances the stability of STAT3 mRNA in an m6A-dependent manner, activating the STAT3 signaling pathway (Sun et al., 2023); in osteosarcoma, ALKBH5 increases SOCS3 expression in an m6A-YTHDF2-dependent manner, thereby inactivating the STAT3 pathway and regulating tumor cell proliferation (Yang Z. et al., 2022). Reader proteins serve as central relays of the pathway, governing its activation as well as tumor progression and metastasis. It has been shown that METTL3 can promote STAT3 protein expression by regulating translation through the m6A-YTHDF1-dependent pathway (Wu et al., 2019); in gastrointestinal tumors, YTHDF2 can degrade IL11 mRNA, inhibiting STAT3 phosphorylation and tumor growth (Hou et al., 2019); it has been shown that the YTHDF3/m6A-EGFR/STAT3 and EMT pathways can stimulate the progression of HCC (Hu B. et al., 2023); YTHDC1 enhances the stability of PIK3R1 in an m6A-dependent manner, thereby mediating STAT3 signaling and regulating the development of ovarian cancer (Wang et al., 2023d); Yang et al. (Yang N. et al., 2020) showed that deletion of YTHDC2 can regulate cancer-related pathways, including p53, NF-κB, and JAK-STAT signaling; ALKBH5/IGF2BP3 upregulates XBP1 expression and activates IL-6-JAK-STAT3 signaling, thereby promoting the proliferation, migration, and invasion of NSCLC cells (Liang et al., 2025); downregulation of HNRNPC can reduce HIF-1α expression and inhibit IL-6/STAT3-mediated HCC metastasis, and is associated with poor prognosis (Liu et al., 2022b); the interaction between HNRNPC and KH-type splicing regulatory protein can induce the invasion and metastasis of human lung cancer cells by activating the IFN-α-JAK-STAT1 signaling pathway (Yan et al., 2019); Gao et al. (2021) demonstrated that the hnRNPA2B1-STAT3-VEGF-A axis plays an important role in breast cancer angiogenesis; Esteves et al. (2020) reported that eIF3f can interact with STAT3 and increase Snail2 expression, thereby promoting lung cancer metastasis.

### 4.9 m6A and HIF

Core m6A methyltransferases are major downstream effectors of HIF-1α under hypoxic conditions, and concurrently modulate hypoxic signaling transduction to drive tumor metabolic reprogramming, metastasis and drug resistance. In lung cancer, HIF-1α can regulate the expression of METTL3, and METTL3-mediated NFE2L3 m6A modification can activate the Wnt/β-catenin signaling pathway, promoting tumorigenesis and drug resistance (Zhao Y. et al., 2025); Hu Y. et al. (2023) demonstrated that UCA1 binds to METTL14 to activate the HIF-1α and NF-κB signaling pathways and regulate the development of psoriasis; in liver cancer, hypoxia can upregulate METTL16 expression through HIF-1α transcription and downregulate the RNA stability of Lnc-CSMD1-7, ultimately promoting HCC metastasis (Wang et al., 2023e); studies have shown that WTAP is regulated by HIF-1α and promotes the Warburg effect in ovarian cancer cells (Lyu et al., 2022); in pancreatic cancer, the VIRMA-STRA6-STAT3-HIF-1α axis plays an important role in tumor glycolysis and malignant progression (Yang K. et al., 2024); in addition, ectopic PSF expression can recruit Hakai to the PSF/HIF-1α complex, thereby inhibiting hypoxia-induced HIF-1α activation in the nucleus (Dong L. et al., 2021); Tang et al. (2024) demonstrated that RBM15 overexpression led to increased expression of GLUT1, hexokinase, HIF-1α, and PFKFB3. Canonical m6A demethylases FTO and ALKBH5 are direct transcriptional targets of HIFs (Xiao et al., 2020), forming a typical hypoxia-responsive m6A regulatory loop to finely tune HIF-1α signaling activity. In esophageal cancer, FTO can increase the stability of LINK-A and subsequently eliminate the MCM3-mediated transcriptional inhibition of HIF-1α (Nan et al., 2023); Zhang et al. (Zhang et al., 2016) reported that breast cancer cells exposed to hypoxia can regulate ALKBH5 expression in a HIF-1α- and HIF-2α-dependent manner. Studies have shown that YTHDF1 can regulate the methylation and expression of HIF genes, thereby promoting the progression of hypoxia-related tumors (Shi Y. et al., 2019); The m6A reader proteins exhibit prominent tumor-specific bidirectional regulation on HIF-1α signaling, controlling hypoxic tumor metabolism, proliferation and metastasis via modulating HIF gene methylation, mRNA stability and translation. In liver cancer, the METTL14/HIF-1α/m6A modification axis can induce SLC7A11 mRNA through the YTHDF2/SLC7A11 pathway, participating in tumor regulation (Fan et al., 2021); in pancreatic cancer, YTHDC1 can promote miR-30d to target the key glycolytic genes HIF-1α and MYC, reducing glycolysis and inhibiting PDAC tumorigenesis (Hou et al., 2021); Tanabe et al. (2016) confirmed that YTHDC2 can target the transcription factor HIF-1α for translation, playing an important role in the metastasis of colon tumor cells; in gastric cancer, IGF2BP3 can upregulate HIF-1α expression by directly binding to a specific m6A site in the coding region of HIF-1α mRNA (Jiang L. et al., 2021); in liver cancer, HNRNPC downregulation can reduce HIF-1α expression by destabilizing HIF-1α mRNA, and HIF-1α overexpression rescues the reduction in HCC invasiveness and metastasis induced by HNRNPC downregulation (Liu et al., 2022b); Soung et al. (2019) demonstrated that hnRNPA2B1 binds to the 3′-UTR of HIF-1α mRNA, enhancing HIF-1α protein expression; Miao et al. (Miao et al., 2019) found that eIF3a is overexpressed in HCC tissues, and its activity is consistent with the activation of genes in the HIF-1α pathway.

### 4.10 m6A and MYC

Methyltransferases act as the dominant positive regulators of MYC, elevating its expression across diverse cancers through a variety of m6A-dependent routes. In prostate cancer, METTL3 mediates the m6A modification of SNHG7 and enhances its stability, thereby regulating c-Myc and the proliferation and glycolysis of tumor cells (Liu et al., 2022e); its regulatory effect on MYC has been confirmed in gastric cancer (Yang D.-D. et al., 2020), prostate cancer (Wu et al., 2021), AML (Vu et al., 2017), thymic epithelial tumors (Iaiza et al., 2021), oral cancer (Zhao et al., 2020), and lung cancer (Wu et al., 2021); in liver cancer, METTL5 can stabilize c-Myc by increasing USP5 translation, thereby promoting the proliferation and metastasis of HCC cells (Xia et al., 2023); METTL14 can enhance the proliferation and migration of cervical cancer cells by regulating the m6A level of the Myc oncogene (Hu C. et al., 2022); Xie et al. (2024) demonstrated that MYC is responsible for the dysregulation of METTL16, and METTL16 can mediate the process by which MYC regulates GLUL; ZCCHC4 participates in the regulation of ESCC cell sensitivity to cisplatin through the ROS/c-Myc axis (Yao et al., 2025); in AML patients, when WTAP is knocked down, MYC mRNA m6A methylation levels decrease, leading to upregulation of c-Myc (Naren et al., 2021); in ovarian cancer (Han et al., 2020) and pancreatic cancer (Cao et al., 2024), WTAP has also been confirmed to be involved in the regulation of MYC; in cervical cancer, RBM15 can bind to c-Myc mRNA, resulting in increased c-Myc protein expression (Nie et al., 2023). Canonical m6A demethylases are indispensable for maintaining MYC stability and oncogenic activity, with FTO exerting pan-cancer regulatory effects on MYC. FTO can increase MYC expression by preventing m6A modification and further promote the progression of CRC (Yue et al., 2020); the role of FTO in regulating MYC has been confirmed in cervical cancer (Zou et al., 2019), lung cancer (Yang X. et al., 2021), gastric cancer (Locasale, 2013), and AML (Su et al., 2018); Qiao et al. (2023) demonstrated that METTL3/FTO/ALKBH5/IGF2BP2 can synergistically regulate the stability of c-Myc mRNA in an m6A modification-dependent manner, promoting esophageal cancer tumorigenesis; m6A reader proteins recognize m6A-modified MYC transcripts and regulate their stability and transcription in a tumor-specific manner, constituting the core downstream execution mechanism of m6A-mediated MYC signaling regulation. In OSCC, METTL3 can enhance the stability of c-Myc through YTHDF1-mediated m6A modification and promote tumor cell progression (Han et al., 2021b); in glioma stem cells, YTHDF2 promotes glioma stem cell progression by stabilizing MYC and VEGF mRNA (Dixit et al., 2021); in pancreatic cancer, YTHDF3 plays an important role in tumorigenesis by recognizing m6A modification in MYC mRNA (Zhang H. et al., 2024); in liver cancer, m6A-modified ATP8B1-AS1 interacts with YTHDC1 and is recruited to the MYC promoter region, resulting in transcriptional activation of MYC (Tan et al., 2023); in bladder cancer (Xie F. et al., 2021), liver cancer (Wei L. et al., 2021), colorectal cancer (Wang Y. et al., 2019), cervical cancer (Zhu and Thompson, 2019), and renal cancer (Zhao B. et al., 2022), the IGF2BP family can regulate MYC; Kim et al. (2003) found that HNRNPC regulates c-Myc mRNA translation in a cell-cycle-stage-dependent manner through IRES binding; in liver cancer, TNFAIP6 interacts with HNRNPC to stabilize c-Myc mRNA and upregulate PKM2 to promote glycolysis (Duan et al., 2024); in renal cancer, c-Myc directly binds to the promoter of hnRNPA2B1 to drive its transcription (Liu et al., 2020); in colorectal cancer, upregulation of eIF3f leads to reprogramming of the SGOC pathway and regulates PHGDH and MYC stability, thereby promoting tumor cell growth (Pan et al., 2023).

### 4.11 m6A and p53

Upstream of p53, the methyltransferases exert context-dependent effects on pathway activation and tumor progression. Studies have shown that METTL3 can enhance the stability of CircGLIS3 and regulate the p53 signaling pathway to promote cell proliferation and invasion (Cheng X. et al., 2024). Sang et al. (Sang et al., 2022) demonstrated that METTL3 and METTL14 exert their oncogenic potential in AML through the MDM2/p53 signaling axis; The canonical m6A demethylase FTO modulates p53 signaling activity via targeted downstream molecular cascades. FTO can target specificity protein 1 and Aurora kinase B, leading to mutation of ataxia telangiectasia mutated, p38 phosphorylation, and p53 dephosphorylation (Zeng X. et al., 2024); The m6A reader proteins are the core downstream executioners of m6A-dependent p53 regulation, with distinct family members exhibiting diverse functional effects on p53 expression and signaling activation. Studies have shown that p53 mRNA is a key target of m6A methylation and YTHDF1, and YTHDF1 can drive the translation of p53 protein (Chang et al., 2025); in rectal cancer, pleckstrin-2 can cooperate with YTHDF2 to regulate the stability of TYMS mRNA and mediate p53/p21 signaling (Zhou et al., 2025); in a study on uveal melanoma, TRIM2 was confirmed to be an m6A modification substrate of YTHDF3, and TRIM2 can ubiquitinate and degrade p53 protein (Li et al., 2026); research has found that YTHDC1 is a major regulator of p53 expression that can bind to the transcription initiation sites of TP53 and other genes involved in the DNA damage response, regulating the p53 signaling pathway (Elvira-Blázquez et al., 2024); in liver cancer, the METTL3-and IGF2BP2-regulated ubiquitin ligase F-box protein 43 can promote p53 degradation, driving tumor cell proliferation and invasion (Zhou H. et al., 2023); studies have found that depletion of VIRMA can impair ribosome biogenesis by inhibiting mRNA decay and triggering a p53-dependent stress response (Wu et al., 2025b); in esophageal cancer, RBM15 can mediate the maturation of pri-miR-3605-5p into mature miR-3605-5p, leading to KRT4 upregulation and activating the p53 signaling pathway (Wang, 2024).

### 4.12 m6A and TGF-β

The methyltransferases act upstream of TGF-β/Smad signaling, influencing tumor progression, drug resistance, immunosuppression, and tissue fibrosis across many malignancies. In gastric cancer, METTL3 and IGF2BP2 can enhance Smad3 protein expression and promote the activation of the TGF-β/Smad pathway (Yuan et al., 2023); in intrahepatic cholangiocarcinoma, loss of METTL5 impairs the m6A modification of 18S rRNA, thereby hindering ribosome biogenesis and inhibiting the translation of G-quadruplex-containing mRNAs enriched in the TGF-β pathway (Dai et al., 2023); in TNBC, RBM15 enhances the resistance of TNBC to paclitaxel by promoting the m6A methylation of tumor necrosis factor receptor superfamily member 9 and inducing M2 polarization of tumor-associated macrophages (Fu et al., 2025); VIRMA can enhance the activation of the TGF-β signaling pathway by methylating its downstream target gene ADAR, thereby participating in tumor immunosuppression and the progression of NSCLC (Shan et al., 2025); in addition, WTAP can regulate FBLIM1 expression in an m6A-dependent manner, and FBLIM1 promotes the activation and fibrosis of LX-2 cells by regulating the TGF-β signaling pathway (Ren et al., 2025). Canonical m6A demethylases participate in TGF-β signaling modulation and tumor progression via m6A-dependent post-transcriptional regulation. In liver cancer, FTO upregulation leads to decreased m6A modification of BUB1 mRNA, thereby increasing BUB1 protein levels through a YTHDF2-dependent pathway and activating downstream TGF-β signaling (Zhang et al., 2025b); m6A reader proteins act as pivotal downstream executors of m6A-dependent TGF-β signaling regulation, controlling tumor metastasis and malignant progression through diverse molecular mechanisms. In prostate cancer, METTL3 and YTHDF1 jointly regulate the m6A methylation of GDF15, a member of the TGF-β superfamily (Jiang et al., 2026); YTHDC1 enhances the TGF-β signaling cascade by promoting the nuclear export and expression of SMAD3, thereby promoting distant tumor metastasis (Tan et al., 2022); in pancreatic cancer, IGF2BP3 recognizes m6A-modified EMP1 mRNA to increase its stability and promote TGF-β activation (Liu L. et al., 2025); HNRNPC can increase the stability and expression of WDR77 and enhance TGF-β expression, mediating the progression of keloid scarring (Zhan et al., 2026).

## 5 Crosstalk among m6A, signaling pathways, and glycolysis

**FIGURE 3:**
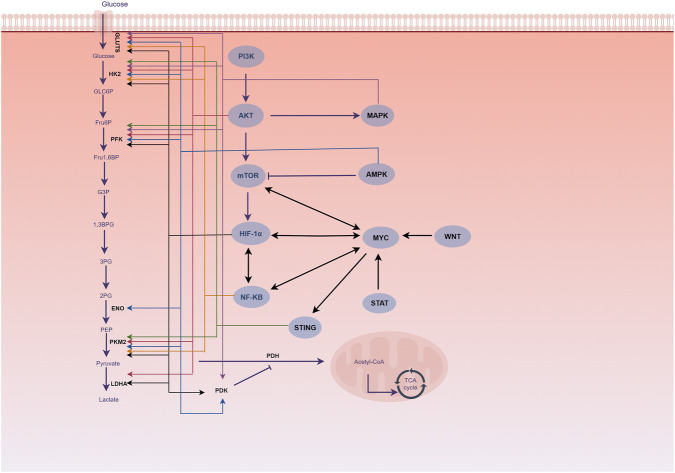
The relationship between signaling pathways and glycolysis regulation.

There is a close inter-regulatory relationship among m6A modification, signaling pathways, and glycolysis, forming a dynamic closed-loop regulatory network that jointly participates in the tumorigenesis and progression of diseases and the regulation of malignant phenotypes (Figure 3). The Wnt signaling pathway can regulate the transcription of various glycolytic enzymes, including LDHA, pyruvate carboxylase, HK2, PFKFB3, and PKM (Xu et al., 2021; Zeng Z. et al., 2024); in lymphoma, NF-κB can promote the translocation of GLUT1 to the plasma membrane (Sommermann et al., 2011); studies have confirmed that STING can target the rate-limiting glycolytic enzyme HK2 to limit aerobic glycolysis and promote anti-tumor immunity (Zhang et al., 2023a); HIF-1α is involved in the production of various enzymes in the glycolytic process, including LDHA, GLUT1, HK2, PDK1, and PFK-1 (Dong et al., 2025); CPSF6 can stabilize the expression of its downstream target c-Myc, thereby enhancing the expression of key glycolytic enzymes such as HK2, PKM2, and LDH (Sim et al., 2024); Emerging studies have validated that the AKT signaling pathway modulates the expression of core glycolytic regulators GLUT1 and PFKFB2, thereby reprogramming cellular glycolysis (Li Z. et al., 2022). Meanwhile, signaling pathways are also regulated by glycolytic enzymes and lactate. Studies have shown that SPOP-mediated degradation of PDK1 suppresses AKT (Jiang Q. et al., 2021); GLUT4 can activate the JAK/STAT signaling pathway to enhance glycolysis in glioma cells (Feng et al., 2022); HK2 can upregulate c-Myc through the Wnt/β-catenin pathway to promote cell cycle progression and drive tumor progression (Liu et al., 2022i); in addition, HK2 can activate the NF-κB signaling pathway (Guo et al., 2022); PKM2 can promote signal transduction at the Y705 site and phosphorylation of STAT3, thereby promoting tumor cell proliferation (Gao et al., 2012); studies have shown that PKM2 exerts positive feedback on the transcriptional activity of the β-catenin, c-Myc, and HIF-1α genes (Luo et al., 2011); PFKP assists in the translocation and transactivation of β-catenin (Lee et al., 2020); LDHA-mediated AKT/mTOR activation promotes the proliferation and invasion of cervical cancer cells (Luan et al., 2021); ENO1 can affect the development and metastasis of CRC by regulating the AMPK pathway (Zhan et al., 2017); Zhang et al. (2022a) confirmed that ENO1 can promote Wnt/β-catenin signaling targets such as β-catenin and c-Myc in skin melanoma; ENO1 acts through the Wnt/β-catenin (Li et al., 2021b), AMPK/mTOR (Shu et al., 2021), and PI3K/AKT (Chen R. et al., 2020) pathways in lung tumor development; lactate secretion enhances IL-8 expression through NF-κB, promoting tumor progression and angiogenesis (Végran et al., 2011); lactate-induced activation of c-Myc increases the expression of the glutamine transporters ASCT2 and SN2, thereby promoting glutamine uptake and catabolism in cancer cells (Pérez-Escuredo et al., 2016); studies have shown that lactate can drive primary tumor growth, chemotherapy resistance, immune evasion, and metastasis through various signaling cascades such as HIF-1α, PKC, cAMP/PKA, and STAT3 (Li X. et al., 2022; Tan S.-M. et al., 2025); lactate has been shown to participate in the regulation of TGF-β/Smad and Wnt/β-catenin signaling pathways and can activate EMT (Niu et al., 2021); Glycolytic enzymes and lactate can also regulate m6A methylation. In tumor cells, accumulated lactate effectively induces the upregulation of METTL3 expression through H3K18 lactylation (Xiong et al., 2022); Yu et al. (2021c) showed that ocular melanoma can use lactate as a substrate for histone lactylation, further promoting YTHDF2 transcription and inducing tumorigenesis; in bladder cancer, GLUT3 upregulates RNF183 expression, thereby destabilizing YTHDC1 (Yan et al., 2023). Numerous studies have shown that multiple signaling pathways can also regulate m6A methylation. In CRC, c-Myc can upregulate YTHDF1 expression, increasing cancer cell proliferation and drug resistance (Nishizawa et al., 2018); in HCC, under hypoxic conditions, HIF-1α can mediate the upregulation of YTHDF1 (Li et al., 2021c); a recent study has shown that METTL14 can be transcriptionally activated by wild-type p53 and inhibits the expression of SLC2A3 and PGAM1, thereby suppressing aerobic glycolysis (Hou et al., 2023). Liu et al. (2022h) confirmed that p53 can transcriptionally regulate ALKBH5 in cancer stem-like cells.

## 6 Conclusion

In summary, m6A modification, as a core mechanism of post-transcriptional RNA regulation, can regulate the expression, activity, and signal transduction of key molecules in various signaling pathways; it can also directly target key glycolytic enzymes and affect the glycolytic process by regulating the methylation status, stability, and translation efficiency of their mRNAs, thereby supporting the metabolic reprogramming of tumor cells. At the same time, signaling pathways can reciprocally regulate m6A modification and glycolysis: on the one hand, multiple signaling pathways can directly regulate the transcription and expression of m6A-related molecules, affecting the overall level and function of m6A modification; on the other hand, signaling pathways can directly participate in the regulation of the glycolytic process by regulating the transcription, translation, and activity of key glycolytic enzymes, promoting the metabolic adaptation of tumor cells. Moreover, glycolysis can also exert feedback regulation on m6A modification and signaling pathways: key glycolytic enzymes and the end product lactate can affect m6A modification levels by regulating the stability, transcription, or translation of m6A-related molecules, and can also activate or inhibit related signaling pathways to further amplify pro-tumor effects, ultimately forming a closed-loop regulatory network in which m6A modification regulates signaling pathways and glycolysis, signaling pathways regulate m6A modification and glycolysis, and glycolysis in turn feedback-regulates m6A modification and signaling pathways.

Based on the current state of research, future studies can be conducted in the following directions. First, the key molecules and core regulatory nodes within the m6A modification–signaling pathway–glycolysis closed-loop regulatory network should be further explored, and the specific molecular mechanisms of their interactions clarified. Second, based on this closed-loop regulatory network, specific inhibitors targeting m6A modification, signaling pathways, or glycolysis should be developed to provide new strategies for the precision treatment of diseases. Third, the association between this closed-loop regulatory network and the tumor immune microenvironment and chemotherapy resistance should be further investigated, and the role of their coordinated regulation in disease progression clarified, providing a theoretical basis for reversing drug resistance and enhancing therapeutic efficacy. Overall, the closed-loop regulatory network of m6A modification, signaling pathways, and glycolysis provides a new perspective for the study of disease pathogenesis and the development of therapeutic targets. In the future, multi-dimensional and interdisciplinary research is needed to further reveal its regulatory rules and to provide more solid theoretical support for the clinical diagnosis and treatment of diseases.
